# The Insect Pest Control Laboratory of the Joint FAO/IAEA Programme: Ten Years (2010–2020) of Research and Development, Achievements and Challenges in Support of the Sterile Insect Technique

**DOI:** 10.3390/insects12040346

**Published:** 2021-04-13

**Authors:** Marc J. B. Vreysen, Adly M. M. Abd-Alla, Kostas Bourtzis, Jeremy Bouyer, Carlos Caceres, Chantel de Beer, Danilo Oliveira Carvalho, Hamidou Maiga, Wadaka Mamai, Katerina Nikolouli, Hanano Yamada, Rui Pereira

**Affiliations:** Insect Pest Control Subprogramme, Joint FAO/IAEA Centre of Nuclear Techniques in Food and Agriculture, Department of Nuclear Sciences and Applications, International Atomic Energy Agency, A-1400 Vienna, Austria; M.Vreysen@iaea.org (M.J.B.V.); A.M.M.Abd-Alla@iaea.org (A.M.M.A.-A.); K.Bourtzis@iaea.org (K.B.); J.Bouyer@iaea.org (J.B.); C.E.Caceres-Barrios@iaea.org (C.C.); C.De-Beer@iaea.org (C.d.B.); D.Carvalho@iaea.org (D.O.C.); H.Maiga@iaea.org (H.M.); W.Mamai@iaea.org (W.M.); K.Nikolouli@iaea.org (K.N.); R.Cardoso-Pereira@iaea.org (R.P.)

**Keywords:** area-wide integrated pest management, autocidal control, plant pests, livestock bests, human disease vectors, genetics and molecular biology, mass-rearing, quality control, radiation, genetic sexing, competitiveness

## Abstract

**Simple Summary:**

The Insect Pest Control (IPC) Section and its associated laboratory (IPCL) is part of the Joint FAO/IAEA Centre of Nuclear Techniques in Food and Agriculture. Its mandate is to develop and implement the sterile insect technique (SIT) for selected key insect pests, and thereby to reduce the use of insecticides, to reduce animal and crop losses, to protect the environment, to facilitate international trade in agricultural commodities and to improve human health. With this aim, the IPCL has been implementing research in relation to the development of the SIT package for insect pests of crops, livestock and human health. This paper provides a review of the research carried out between 2010 and 2020 at the IPCL.

**Abstract:**

The Joint FAO/IAEA Centre (formerly called Division) of Nuclear Techniques in Food and Agriculture was established in 1964 and its accompanying laboratories in 1961. One of its subprograms deals with insect pest control, and has the mandate to develop and implement the sterile insect technique (SIT) for selected key insect pests, with the goal of reducing the use of insecticides, reducing animal and crop losses, protecting the environment, facilitating international trade in agricultural commodities and improving human health. Since its inception, the Insect Pest Control Laboratory (IPCL) (formerly named Entomology Unit) has been implementing research in relation to the development of the SIT package for insect pests of crops, livestock and human health. This paper provides a review of research carried out between 2010 and 2020 at the IPCL. Research on plant pests has focused on the development of genetic sexing strains, characterizing and assessing the performance of these strains (e.g., *Ceratitis capitata*), elucidation of the taxonomic status of several members of the *Bactrocera dorsalis* and *Anastrepha fraterculus* complexes, the use of microbiota as probiotics, genomics, supplements to improve the performance of the reared insects, and the development of the SIT package for fruit fly species such as *Bactrocera oleae* and *Drosophila suzukii*. Research on livestock pests has focused on colony maintenance and establishment, tsetse symbionts and pathogens, sex separation, morphology, sterile male quality, radiation biology, mating behavior and transportation and release systems. Research with human disease vectors has focused on the development of genetic sexing strains (*Anopheles arabiensis*, *Aedes aegypti* and *Aedes albopictus*), the development of a more cost-effective larvae and adult rearing system, assessing various aspects of radiation biology, characterizing symbionts and pathogens, studying mating behavior and the development of quality control procedures, and handling and release methods. During the review period, 13 coordinated research projects (CRPs) were completed and six are still being implemented. At the end of each CRP, the results were published in a special issue of a peer-reviewed journal. The review concludes with an overview of future challenges, such as the need to adhere to a phased conditional approach for the implementation of operational SIT programs, the need to make the SIT more cost effective, to respond with demand driven research to solve the problems faced by the operational SIT programs and the use of the SIT to address a multitude of exotic species that are being introduced, due to globalization, and established in areas where they could not survive before, due to climate change.

## 1. Historical Background

More than 10,000 insect and mite pests are known to affect livestock, human health and crops, both pre- and post- harvest; these species are often at the root of food insecurity throughout the world [1]. The Food and Agriculture Organization of the United Nations (FAO) estimated that in 2015, 815 million people were chronically undernourished worldwide [2] with a prevalence of 18.6%, 16.3%, 10.2%, and 7.6% in sub-Saharan Africa, South Asia, East Asia and the Pacific, and Latin America, respectively [3]. Since Paul Müller discovered the acute toxic properties of the chemical dichlorodiphenyltrichloroethane, better known as DDT, just before the Second World War [4], insect pests have, in the following decades, mainly been controlled using broad-spectrum insecticides. In 2020, 3.5 million tons of pesticides were sprayed worldwide at a total cost of more than Euro 60 billion [5]. This massive and often indiscriminate use of toxic chemicals has raised concerns about residues in food commodities, increased resistance of many pest insects to insecticides, contamination of the environment and outbreaks of secondary insect pests [6]. This epitomizes the urgent need for sustainable insect pest management tactics that are not only effective, but more friendly or neutral to the environment. The sterile insect technique (SIT), when used on an area-wide basis, is such an environment-friendly control tactic that has been used with great success against selected key insect pests that affect crops, livestock and human health [7].

## 2. The Insect Pest Control Laboratory of the Joint FAO/IAEA Centre of Nuclear Techniques in Food and Agriculture: Mandate and Objectives

The FAO was founded in 1945 with the mandate to raise the levels of nutrition and standards of living of human populations under its various jurisdictions, to secure improvements in the efficiency of the production and distribution of all food and agricultural products, to better the condition of rural populations and therefore to contribute to expanding the world economy and ensuring humanity’s freedom from hunger [2].

On 8 December 1953, United States President Dwight D. Eisenhower delivered his “Atoms for Peace” speech to the United Nations General Assembly in New York. He described the potential danger of nuclear war but also emphasized peaceful nuclear applications in medicine, agriculture and power generation. Eisenhower proposed the creation of an “International Atomic Energy Agency” (IAEA) to be set up under the aegis of the United Nations. It took four years of negotiations to bring the IAEA into being and to reach agreements regarding the organization’s mandate, to negotiate the statute and to decide on upon a location (https://www.iaea.org/about/history/atoms-for-peace-speech, accessed on 10 February 2021).

Initially, there were overlaps between the two organizations with respect to nuclear energy, i.e., the FAO had an Atomic Energy Branch, and the IAEA established an Insect Pest Control Section in 1960 in response to requests from member states following the successful start of the New World screwworm SIT program in Florida, and in 1961, a Unit of Agriculture at its Laboratories. To address these duplications, the Director General of the two organizations reached an agreement on a joint approach to promote nuclear techniques in food and agriculture which culminated in 1964 in the establishment of the Joint FAO/IAEA Division of Atomic Energy in Agriculture (later renamed to Joint FAO/IAEA Centre of Nuclear Techniques in Food and Agriculture) with the relevant FAO staff and resources in this area moving to Vienna. In 1958, the IAEA General Conference recommended the establishment of laboratories in Austria, and in 1961, the IAEA laboratories were inaugurated. The mandate of the laboratories is to support the activities of the Joint FAO/IAEA Centre through training, research and services [8].

The Joint FAO/IAEA Centre is not only unique in the UN system in view of its research laboratories (and coordinated research projects (CRPs)), but also because both FAO and IAEA contribute funds and staff salaries, and approve the work program and budget every two years. There is also an oversight committee with representatives from both FAO and IAEA that meets once a year.

The Joint FAO/IAEA Centre is currently composed of five sections, one of which deals with insect pest control, with the mandate to assist member states with area-wide integrated pest management (AW-IPM) of major insect pests of crops, livestock and human health through developing and integrating the SIT with other control methods. The programmatic objectives of the insect pest control subprogram are to reduce the use of insecticides, to reduce animal and crop losses, to facilitate international trade in agricultural commodities, to protect the environment and to improve human health.

In the first decades after its founding, the focus of the Insect Pest Control Laboratory was the development of the SIT for fruit flies and tsetse flies, and later Lepidoptera (through CRPs) and, finally, mosquitoes since 2003.

## 3. Area-Wide Integrated Pest Management and the Sterile Insect Technique

Sixty years ago, E.F. Knipling [9] emphasized the importance of applying insect pest control techniques on an area-wide basis, targeting the entire pest population within a defined area, as opposed to a field-by-field approach where portions of the pest population are left untouched [10].

**Figure 1:**
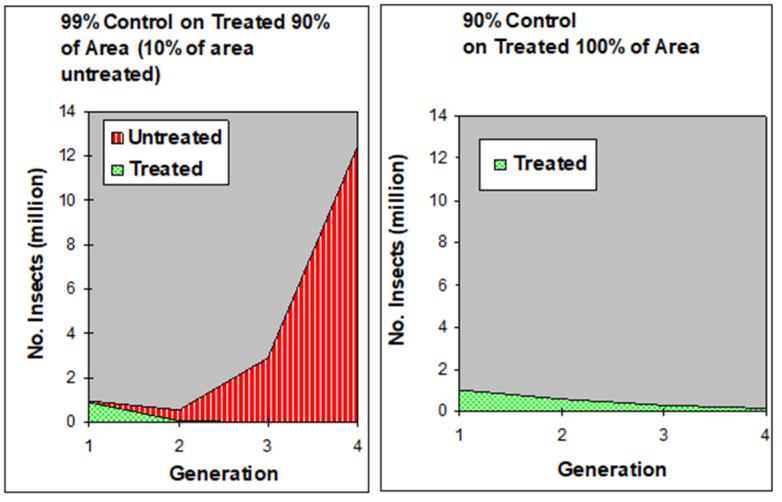
Results of a model that shows the outcome of neglecting to suppress a small fraction of a pest population in an agroecosystem versus the effect of uniformly suppressing the entire pest population. Left: 10% of the population is untreated, and in four generations it produces a large number of individuals, while the 90% of the population that is treated declines. Right: Entire pest population in the agroecosystem is suppressed uniformly, and its numbers decline from generation to generation (Figure from Klassen and Vreysen, 2021, reproduced with permission).

Using simple mathematical models, Knipling [11] compared two classic scenarios: in the first, 99% of the population was destroyed every year but only in 90% of the area (i.e., the remaining 10% was left untouched) while in the second, only 90% of the pest population was eliminated but from the total area (100%) each year. The model indicated that after five years, the insect population would be 100 times higher in the first area than in the second (Figure 1). Therefore, imposing less intensive suppressive pressure against the total pest population is more effective than subjecting only part of the population to more intensive control efforts [10].

Although sterile insects were used for the first time in 1954 to manage an insect pest population, the concept had been conceived 20 years earlier [12]. This innovative idea represented a complete shift in insect pest control techniques, i.e., rather than affecting the survival of insect pest populations through the use of insecticides [13], removal of hosts [14,15], or changing the ecosystem (habitat destruction) critical for the survival of the insect [16], SIT compromises the hereditary machinery of insect pest populations [9].

Like any control tactic, the use of sterile insects has its limitations and advantages. The technique requires a substantial initial financial investment to establish an insect rearing facility. Ionizing radiation is used to sterilize the released insects, which should be released on an area-wide basis to avoid immigration of gravid females into the target area. Additionally, it is not a stand-alone technique, requiring, in most cases, prior suppression of pest populations [17]. It also requires coordination among all stakeholders, long-term commitment, usually multiyear planning and a centralized organization dedicated exclusively to its implementation [18]. In addition, sterile insects have no immediate impact on the pest population as, contrary to other control tactics, they do not directly kill the insects in the field, but introduce sterility in the target population. As such, the effects are only seen in subsequent generation(s) [19].

The main strength and uniqueness of the SIT relates to its action that is inversely dependent on the density of the target population [20], i.e., the effectiveness of the method increases as the pest population declines in number. Initial releases of a given number of sterile insects will result in a decline of the population in the first generation, and if this is followed with continued releases at the same rate, the sterile to wild male ratios will increase with each generation [17]. In addition, the SIT is species specific, in that it exerts its effect through con-specific matings of released sterile insects and their wild counterparts. The release of sterile insects is friendly to the environment, as it reduces the use of insecticides and, unlike other biological control tactics (e.g., parasitoids), released insects cannot become established [21].

It needs to be emphasized that not all insect pests are amenable to be treated with sterile insects. The technique is only suitable for pests where the stage that is released, usually the adult, does not contribute to the damage to crops or livestock, or some insect vectors of diseases (e.g., horn flies), species where the adults are plant herbivores (e.g., locusts) and species where adults cause nuisance (e.g., house fly, cockroaches). Other prerequisites are: (1) the need for a comprehensive knowledge of the ecology and behavior of the insect, (2) the colonization and mass-production of the target insect should be feasible at reasonable cost, (3) the population density can be reduced using economically feasible and ecologically acceptable suppression techniques, (4) the competitiveness of the reared and released insects should be as close as possible to that of their wild counterparts, and (5) the sperm of the sterilized males should be as competitive as the sperm of the wild insects [19].

## 4. Main Research Achievements—Plant Pests

### 4.1. Genetic Sexing Strains

Although successful AW-IPM projects with an SIT component have been implemented using bisexual strains, several studies have clearly indicated that the efficacy and cost-effectiveness of SIT can be greatly enhanced by using genetic sexing strains (GSS) which would allow male-only releases [22,23]. The IPCL has been at the forefront of the development of successful GSS for the Mediterranean fruit fly *Ceratitis capitata*, known as VIENNA GSS [23]. A number of translocation lines have been developed over the years; in the last 20 years, two of them have been used in AW-IPM programs with an SIT component worldwide: the VIENNA 7 GSS, which carries the T(Y;5)58B translocation (also known as T(Y;5)3-129 strain) and the VIENNA 8 GSS, which carries the T(Y;5)52A translocation (also known as T(Y;5)101 or T(Y;5)30C strain) [24]. The VIENNA 8 GSS is available in two versions, with and without the inversion D53 (VIENNA 8 and VIENNA 8^D53^ GSS). This inversion covers a significant part of chromosome 5, which is the chromosome where the translocation breakpoints and the two selectable markers of the GSS, the *white pupae* (*wp*) and *temperature sensitive lethal* (*tsl*) genes, are located [25], thus suppressing the recombination and improving the generic stability of the strain [23,24]. After many years under mass-rearing conditions, with an average weekly output of about 2000 million sterile males, VIENNA 7 and VIENNA 8 strains from all facilities worldwide were evaluated with respect to their “sexing” characters and genetic stability, which are based on the *white pupae* and *temperature sensitive lethal* genes, as well as their overall biological quality. The quality control evaluation, as well as cytogenetic and mitochondrial DNA analyses, indicated that these strains were extremely stable. However, despite their common origin, they presented differences with respect to rearing efficiency and thermotolerance which require further investigation [26].

The development and the monitoring of the stability of GSS requires a thorough cytogenetic analysis and good knowledge of their mitotic and polytene chromosomes [24]. Such cytogenetic analyses of the mitotic and polytene chromosome, as well as the characterization of the translocation breakpoints, were carried out not only for *Ceratitis capitata* GSS, but also for *Bactrocera dorsalis*, *Zeugodacus cucurbitae*, *Anastrepha ludens* and *Anastrepha fraterculus* sp. 1 [24,27,28,29,30].

### 4.2. Ceratitis Capitata

The VIENNA GSS is based on a reciprocal translocation, that comprises the Y chromosome, and autosome 5, which carries two selectable markers, the *white pupae* (*wp*) and the *temperature sensitivity lethal* (*tsl*) genes. Male flies emerge from brown pupae and are resistant to high temperatures, whereas female flies emerge from white pupae and are sensitive to high temperatures (32–34 °C), making elimination of the females possible at the embryonic stage. The females also develop more slowly as larvae as compared with male flies [23,31]. Recent studies showed that the slower development of the female larvae is due to a genetic locus, namely *slow development* (*sd*), which is closely linked to the *tsl* gene. By exploiting genetic recombination phenomena, albeit at a low rate, between the *tsl* and *sd* loci, a new *tsl* strain was developed exhibiting faster developing female larvae (tsl-FD) in comparison with the previous *tsl* strain. The introgression of the tsl-FD into the VIENNA 8^D53^-GSS resulted in a new strain, the “VIENNA 8^D53^-FD GSS”. Characterization of this new GSS confirmed that female larvae develop faster, but also revealed differences in the temperature sensitivity and production profile [32]. The better biological profile of the new tsl-FD GSS strain will increase the rearing efficiency of *C. capitata* SIT projects.

Available GSSs of the Mediterranean fruit fly and other fruit flies have mostly been developed using classical genetics [23]. However, molecular genetics approaches have also been used to develop transgenic sexing strains (TSSs) that are based on female embryonic lethality. One of these strains, FSEL#32 TSS, was developed at the Institute für Zoologie und Entwicklungsbiologie, Georg-August-Universität Göttingen, Germany. When supplied with tetracycline, it produces males and females. Removing tetracycline from the diet results in only male progeny, as the specific female lethality is driven by the absence of this compound [33]. The strain was compared with the VIENNA 8^D53^- GSS [23] in terms of production and quality control profile. The FSEL #32 TSS showed a good production profile, and the sexing mechanism proved very stable, but the quality control profile of the VIENNA 8 GSS was better, especially with respect to survival under starvation, but flight ability and mating competitiveness were similar for the two strains [30]. Although the FSEL#32 TSS shows potential for SIT projects, the strain needs to be evaluated under mass-rearing conditions, and its performance during open field releases has to be assessed.

Production parameters and mating behavior of another transgenic strain, VIENNA 8–1260, that expresses Ccb2t promotor driver tGFP in the testes and DsRed in the body, was compared with two other VIENNA GSS, the VIENNA 8 and the VIENNA 8 Sr^2^ that carries, in addition to the *tsl* and the *wp*, another visible marker. VIENNA 8–1260 produced significantly fewer eggs than the two other strains, but egg hatch was similar. Sterility induced in females of the three strains, after having mated with males irradiated with 100 Gy as pupae, was also different. Differences in male mating competitiveness were gradually reduced with progressing generations [34].

It needs to be mentioned that in some countries, regulations for the utilization of transgenic organisms could be a constraint.

### 4.3. Wolbachia-Infected VIENNA-8 Genetic Sexing Strain

*Wolbachia pipientis*, a maternally inherited obligatory intracellular symbiont, has been reported in several insect species. Infected insects undergo alterations in their reproductive capacity; mating of a *Wolbachia*-infected male with a noninfected female induces cytoplasmatic incompatibility (CI) that causes embryonic lethality. Two lines of *C. capitata* artificially infected with two strains of *Wolbachia* (*w*Cer2, *w*Cer4) were considered as potential strains for use in an incompatible insect technique (IIT) [35] strategy to manage populations of *C. capitata* in an environment-friendly way. Laboratory and walk-in field cage assessments (Figure 3) with *Wolbachia*-infected and noninfected VIENNA 8 GSS showed some differences between the tested strains, indicating that *Wolbachia* infection may negatively affect parameters such as fecundity, male mating competitiveness, adult flight ability and longevity under food deprivation [36]. These results should be taken into consideration when selecting a strategy of IIT alone or in combination with SIT to control *C. capitata*.

### 4.4. Anastrepha ludens and Anastrepha fraterculus

**Figure 2:**
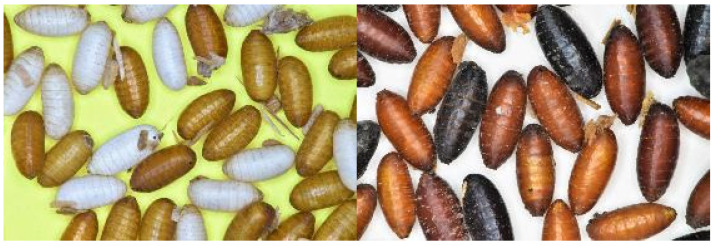
Left: Brown pupae (males) and white pupae (females) from the *Ceratitis capitata* VIENNA 8 GSS. Right: Brown pupae (males) and black pupae (females) from the *Anastrepha ludens* Tapachula 7 GSS (Photocredit: C. Caceres).

For several decades, the SIT has been used as the main control tool to eradicate the Mexican fruit fly *Anastrepha ludens* from California and Texas, USA, and to suppress populations in commercial orchards in northern Mexico [37]. To increase the efficiency of SIT to control the Mexican fruit fly, a GSS (Tapachula-7) was developed by inducing a chromosomal translocation between the “Y” chromosome that carries the maleness factor and chromosome 2 that carries the black pupae locus (*bp*), which determines the color of the pupae. Males are heterozygous, expressing the wild type, brown pupae phenotype, while females are homozygous for the mutant allele and express the black pupae phenotype (Figure 2) [28]. A mechanical-optical sorter can separate the pupae by color, allowing the SIT programs in the USA and Mexico to implement male-only releases [38].

More recently, a new mutation was discovered that slows larval development. The gene responsible for this phenotype (*sl*) is also located on chromosome 2, and hence, is linked to the black pupae locus (*bp*) [39]. Linking the *sl* and *bp* mutations allowed the development of a new GSS in which females are homozygous for the mutant alleles exhibiting black pupae color and slow larval development phenotype. The larvae of the heterozygous males will develop one or two days faster than those of females, which allows the collection of almost exclusively males during the first larvae/pupae collection. In case there are any black pupae escapers, these can be efficiently separated using a mechanical optical sorter.

In South America, the fruit fly *Anastrepha fraterculus* is a major agricultural pest of several fruits and vegetables of economic importance. Phytosanitary regulations have caused trade restrictions of agricultural commodities between infested and not infested regions or countries. The SIT could be an additional component to control this pest on an area-wide basis. Similarly to the GSS developed for *A. ludens*, a GSS based on a pupal color dimorphism (brown-black) was developed to allow male-only releases [40]. Several GSS lines were constructed by the induction of reciprocal translocations that involved the Y chromosome and the autosome carrying the wild type allele. One GSS line was already selected for evaluation under mass-rearing conditions and subsequent field evaluation in Brazil [40]. The GSS lines were constructed with the *A. fraterculus* morphotype 1 that is distributed in Southern Brazil and Argentina, and hence, sterile male-only releases will be feasible in the infested areas of these two regions.

### 4.5. White Pupae Bactrocera Introgressed Line

A *Bactrocera* introgressed white pupae line (BIL) was developed by crossing wild type *B. tryoni* males with white pupae *B. dorsalis* females (parental cross) followed by nine consecutive crosses and backcrosses between wild type *B. tryoni* males with BIL females. Genome sequencing of the *B. tryoni* and the BIL lines indicated that about 99.2% of their genes are of the same (tryoni) origin, with the majority of the *B. dorsalis* genome having been removed during the backcrosses [41]. Therefore, within the same species, introgression may be used as routine practice in SIT programs to refresh the genetic background of laboratory adapt strain. That practices may make it possible to maintain the quality and sexual competitiveness of the sterile males released in the field.

### 4.6. Cryopreservation

In addition to several genetic sexing strains, the IPCL currently maintains over 150 different strains and populations from over 15 different fruit fly species. To reduce the labor costs and economize on space, efforts were made to develop cryopreservation approaches for the long-term storage of these important strains. A standard operating procedure was developed which allowed the cryopreservation of VIENNA 8 GSS without any negative impact on its “sexing” characteristics, genetic structure and stability, or on rearing efficiency and biological quality as determined under both small- and large-scale rearing conditions [42,43].

### 4.7. Species Complexes

As the SIT is species-specific, the genetic relationship among members of species complexes needs to be elucidated. A multidisciplinary approach was used to elucidate the taxonomic status of five members of the *Bactrocera dorsalis* complex, i.e., *B. dorsalis*, *Bactrocera philippinensis*, *Bactrocera invadens*, *Bactrocera papayae*, and *Bactrocera carambolae*, all collected from different geographical areas. Comparative morphological analyses, mating compatibility as assessed in walk-in field cages, cytogenetic analysis, and phytosanitary cold treatments contributed to determine species boundaries and resulted in the synonymization of *B. dorsalis*, *B. philippinensis*, *B. invadens* and *B. papayae* [44,45,46,47]. This has far reaching implications for the elimination of trade barriers between countries that were infested with assumed different species and for the implementation of SIT projects.

Mitotic and polytene chromosome analysis of a *Ceratitis fasciventris* F2 population, a member of *Ceratitis* FAR complex, was also carried out [48]. In an effort to provide additional tools for the resolution of relationships among members of species complexes, the mitogenomes of *B. dorsalis*, *B. carambolae* and *C. fasciventris* F2 populations were characterized and can be used in genotyping studies [48].

Mating compatibility studies in walk-in field cages between *A. fraterculus* populations from Argentina, Brazil, Colombia and Peru provided evidence of postzygotic isolation that triggered the hypothesis of the existence of a cryptic species complex in *A. fraterculus* [49]. Evidence from integrative taxonomy, pre- and post- zygotic isolation studies, male pheromone analyses, cytogenetic analyses, morphological and symbiont characterization confirmed this hypothesis [50]. Complete resolution of the cryptic species for *A. fraterculus* is still ongoing, but it is clear that these results will have a serious impact on SIT programs.

In addition, the role of *Wolbachia* was investigated in the *A. fraterculus* species complex. *Wolbachia* infection was molecularly characterized in the morphotypes Brazilian-1 and Peruvian [51,52]. Mating experiments showed that the symbiont induces unidirectional incompatibility in each of the two morphotypes. However, bi-directional incompatibility was not observed, suggesting that *Wolbachia* is not the main factor in the mating isolation observed between these two morphotypes [52].

### 4.8. Microbiota and Probiotics

Through evolution, insects have developed advanced symbiotic associations with diverse microorganisms, mainly with bacterial species, which play a catalytic role in several components of their biology including, but not restricted to, nutrition, immunity, behavior and reproduction [53]. During the last decade, culture-dependent and culture-independent approaches were used to characterize the symbiotic associations of insect pest species such as fruit flies, tsetse flies and mosquitoes with the ultimate goal of harnessing them for the enhancement of mass rearing SIT applications [53,54,55,56].

Augustinos et al. [57] isolated three gut-associated cultivable bacterial species of the *C. capitata* VIENNA 8 GSS, i.e., *Enterobacter* sp., *Providencia* sp. and *Acinetobacter* sp. The *Enterobacter* sp. AA26 isolate was initially used as a probiotic (and/or protein) supplement to the larval diet; it significantly improved the rearing efficiency through increased pupal and adult recovery rates (productivity) and by reducing the egg-to-adult developmental duration, in particular for males. However, there was no effect on other important traits such as pupal weight, sex ratio, longevity under starvation, flight ability and male mating competitiveness. In a follow-up study, the gut-associated symbionts *Enterobacter* sp. AA26 and *Klebsiella oxytoca* of *C. capitata* were tested as probiotics in both larval and adult diet, and the data showed that: (a) in contrast to *Enterobacter* sp. AA26, *K. oxytoca* does not improve the pupal and adult recovery rate; (b) *K. oxytoca* has a positive impact on the egg-to-adult developmental duration, and on flight ability and (c) when provided as dietary supplements at the adult stage, neither *Enterobacter* sp. AA26 nor *K. oxytoca* had an impact on male mating competitiveness [58]. Based on these data, the biokinetic properties and nutritional value of *Enterobacter* sp. AA26 was further evaluated and tested as dietary supplement (protein source) for its ability to replace brewer’s yeast in the larval diet of *C. capitata* [59,60]. The results of these experiments clearly indicated that yeast can be replaced either partially or fully by *Enterobacter* sp. AA26. Moreover, the addition of this gut-associated symbiont to the larval diet improved productivity, accelerated development, increased pupal weight and enhanced survival under stress conditions without affecting sex ratio, fecundity, flight ability or male mating competitiveness [60].

Culture-independent 16S *rRNA* gene-based next generation sequencing approaches have shown that the structure of gut-associated microbiota of *Bactrocera oleae*, *Anastrepha grandis*, *A. ludens* and *A. fraterculus* (sp.1 and the Andean lineage) are species-specific and depend on the development stage, the diet and on the degree of domestication [61]. An analysis indicated that the gut-associated bacterial communities are mainly dominated by members of Enterobacteriaceae, and mainly by *Enterobacter* and *Providencia* species, while *Morganella* species seem to be only associated with *B. oleae*.

*Candidatus* Erwinia dacicola is the dominant symbiont of *B. oleae* which seems to be lost during domestication, raising the question of whether this loss can be potentially replaced by other gut-associated bacterial species of the same or different tephritid species [62,63]. To test this hypothesis, Koskinioti et al. [63] evaluated seven gut-associated bacterial species, four isolated from *B. oleae* and three isolated from *C. capitata* (*Enterobacter* sp. 23, *Providencia* sp. 22, *Bacillus* sp. 139, *Serratia* sp. 49, and *Enterobacter* sp. AA26, *Providencia* sp. AA23 and *K. oxytoca,* respectively) for their potential as olive fruit fly larval diet supplements. The data suggested that *Enterobacter* sp. AA26, isolated from *C. capitata*, could be potentially used as diet supplement for *B. oleae* as it reduced developmental time, improved productivity and increased pupal weight.

The same seven gut-associated bacterial species were also evaluated for their potential to improve the rearing of *Diachasmimorpha longicaudata*, a parasitoid of several tephritid species, including *B. oleae* and *C. capitata*. The seven bacterial isolates were added to the diet of *C. capitata* larvae which were used as hosts for the development of the parasitoid. The results indicated that *Enterobacter* sp. AA26 bacteria, both live and autoclaved, improved fecundity, parasitism rate and female production, and reduced the developmental time required for adult emergence, while they had no effect on the sex ratio of the parasitoids [64]. Taken together, these data suggest that this gut-associated symbiont, the same as was shown to have positive effects on Mediterranean fruit fly and olive fruit fly rearing, also has the potential to improve the rearing of the parasitoid *D. longicaudata* [64].

### 4.9. Tephritid Genomics and Functional Genetics in Support of SIT Applications

During the last few years, several initiatives have aimed at characterizing the genome of tephritid species and exploiting this in support of SIT applications. Staff of the IPCL participated in an effort to sequence the genome of *C. capitata* and *B. oleae* [65,66]. One of the goals was to characterize the associated microbiome and to detect potential horizontal gene transfer events, that is, bacterial genes which may have been integrated into the insect host genome. In contrast to what has been observed in other insect species, no clear evidence of horizontal gene transfer events was detected [65,66]. The second goal was to obtain good reference genomes of SIT target species which would facilitate future molecular and functional genetics studies for: (a) the characterization of the sex determination pathway in tephritid pest species and the isolation of the gene encoding for the male determining factor (also known as maleness (M) factor) and (b) the identification of the genes encoding for traits which have been (or could be) used as selectable markers in the construction of GSS including, among others, the *white pupae* and *temperature sensitive lethal* genes.

Indeed, future studies successfully isolated both the M factor and the *white pupae* gene. Meccariello et al. [67] used an integrated approach combining comparative genomics and transcriptomics, and functional genetics and initially identified the M factor, which is responsible for determining the male sex in *C. capitata*. This intronless gene was named *Maleness-on-the-Y* (*MoY*). It is located on the long arm of the Y chromosome and encodes a small protein which is necessary and sufficient for male development. *MoY* orthologs were discovered in other tephritid species including *Bactrocera jarvisi*, *B. oleae*, *B. dorsalis*, and *B. tryoni*. The functional conservation of the *MoY* gene in tephritids was confirmed by carrying out RNAi experiments in *B. oleae* and *B. dorsalis* which resulted in feminization of XY insects [67].

The pupal color morphological marker has been used for the development of GSS in three major tephritid pest species, *C. capitata*, *B. dorsalis* and *Z. cucurbitae*. In these GSS, males emerge from brown pupae while females emerge from white pupae, and they are currently used in SIT applications worldwide. Despite almost 40 years of use of this trait as a selectable marker, the gene responsible for this phenotype had remained unknown until recently. In a recent study, [41] employed an integrated approach based on genetics, cytogenetics, comparative genomics and transcriptomics, gene editing and bioinformatics, to isolate the *white pupae* (*wp*) gene which is located on polytene chromosome 5 and is responsible for the pupae color in tephritids. In all three species, the wild type color of pupae is brown, while different, parallel causal mutations in the *white pupae* gene render the puparium white [41]. Using CRISPR/Cas9 gene editing approaches novel *white pupae* mutations were developed in *C. capitata*, while white pupae mutant strains were developed for the first time in *B. tryoni*, which opens the way for the construction of GSS in support of SIT application against this pest species in Australia [41].

The discovery of the male determining genes in tephritids and other pest and disease vectors, as well as of the *white pupae* gene, which is present in diverse insect species, paves the way for the development of a generic approach for the construction of genetic sexing strains. CRISPR/Cas9-based approaches can be used to induce mutations in *white pupae* orthologs and establish mutant lines in species of interest. CRISPR/Cas9-based approaches can also be designed to insert the wild type (rescue) allele of the *white pupae* gene (or of any other suitable selectable marker) in the male determining region. This will result in a GSS in which males will be wild type, while females will be expressing the mutant phenotype. It is important to note that such GSS will be nontransgenic (no foreign DNA will be inserted) and, also importantly, will have negligible risk of genetic instability since the rescue allele will be closely linked to the male determining region.

### 4.10. Nutritional, Hormonal and Semio-Chemical Supplements

Supplying fruit flies with a mixture of protein and sugar accelerates the development of their reproductive organs; hence, they become sexually mature at a much younger age [68]. Combining a juvenile hormone (methoprene) treatment with a protein diet for *A. ludens, A. suspensa* and *A. obliqua* adults was more effective compared with supplying protein alone [69]. The topical application of 5 µg of methoprene to *Zeugodacus cucurbitae* males that were prefed with protein not only accelerated their sexual maturity, but also made them more competitive [70]. This technology could be introduced into SIT action programs to improve its efficiency against *Z. cucurbitae*.

Most *Zeugodacus* and *Bactrocera* species are attracted to the natural substances of methyl eugenol (ME) and raspberry ketone (RK) [71,72]. *Bactrocera dorsalis* males that ingest ME can synthesize and convert it into pheromonal components to attract females [71]. These components therefore substantially improve the mating competitiveness of males when compared with ME-deprived males. Sterile males pretreated with ME not only compete better with wild males for mating with wild fertile females, but are also less attracted to the ME bait stations used for male annihilation technique (MAT), thus opening the possibility of using the MAT and the SIT simultaneously [73,74].

The effective supply of ME to males of *Bactrocera* species is only possible when they are reaching sexual maturity and can respond to these semio-chemicals. Providing the ME to sterile males through feeding [75] becomes complicated at emergence centers and holding facilities, where it is necessary to treat millions of insects per day. To overcome this constraint, the use of aromatherapy treatment was explored, similar to the aromatherapy treatment that is routinely used in SIT programs to enhance the mating performance of sterile *C. capitata* males. Before being released, the males were exposed to the fumes of ginger root oil (apha-copain) [76].

Sterile *B. carambolae* males that were exposed to a ME aromatherapy treatment had a similar mating competitiveness index to males that had ingested ME through feeding. Both types of males, treated either with ME aromatherapy or ME feeding, showed superior mating performance as compared with control males that fed only on sugar. ME aromatherapy is a simple technique that could be implemented in SIT male-only release programs [70].

### 4.11. Development of the SIT Package for Bactrocera oleae

For several decades, research was conducted at the IPCL to develop the SIT package for the olive fruit fly *B. oleae*. However, the main constraints were the lack of consistent rearing outputs and uncertainty with respect to sexual compatibility of a laboratory-adapted strain with olive fruit fly populations from geographical different areas.

To address these issues, an improved egg production system was developed that can be used either in experimental small cages or bigger cages for mass-production. The system consists of a flat wax panel as one of the sides of the adult holding cage. Females oviposit through the wax panel, allowing the collection of eggs outside of the cages which results in an efficient and cost-effective system which may facilitate the implementation of the industrial production of sterile olive fruit flies for SIT application [77].

Second, mating compatibility tests in walk-in field cages were conducted between a laboratory strain (a hybrid with biological material from Greece and Israel) versus wild populations from Croatia, France, Italy and Spain. The results indicated the complete absence of mating barriers between the different populations. These results reinforce the hypothesis that only one laboratory strain can be used to supply sterile insects to any region/county for SIT application to control the olive fruit fly [77].

### 4.12. Development of the SIT Package for Drosophila suzukii

The spotted wing drosophila, *Drosophila suzukii*, is an invasive species which represents a global threat for a wide variety of soft fruit crops. It has been proposed to integrate the SIT in an AW-IPM approach for this pest in greenhouses and other well-confined or isolated areas [78].

Efforts were made to develop a practical and economically viable rearing system including an appropriate egg production and collection device, a suitable larval medium and an efficient pupae collection and pupation system. A functional artificial, cost-effective oviposition system was developed that consisted of two layers of netting that were attached to one of the vertical sides of a rectangular holding cage (30 × 30 × 40 cm). The internal netting had a hole size of about 1 mm^2^ and the external layer of black netting had a hole size of 0.22 mm^2^. Both nets were coated with a fine layer of a wax-paraffin mixture so that females could punch out the solid fine waxed panel with their ovipositor to oviposit the eggs. The eggs remained fixed to the exterior side of the wax layer from where they could be gently collected by washing the waxed panel with water [79]. This novel collection method is similar to the one used for tephritid fruit flies and will greatly facilitate the implementation of *D. suzukii* mass-rearing.

Irradiation dose-response studies indicated that a dose of 90 and 220 Gy under hypoxia and 75 Gy and 220 Gy under normoxia conditions induced 100% and 99.8% sterility in females and males, respectively. A dose of 220 Gy did not affect the adult emergence rate and the flight ability quality control index in both sterile males and females [80].

Nikolouli et al. [78] proposed a complementary approach based on the combination of SIT and the *Wolbachia*-induced IIT. The advantage SIT/IIT can potentially offer for this pest is the low irradiation dose, that confers full sterility in females, while the sterility in males is induced by both the *Wolbachia* infection and the low irradiation dose. This concept was investigated by Nikolouli et al. [81] using two lines of *D. suzukii* infected with different *Wolbachia* strains, *w*Ha and *w*Tei. The analysis showed that a dose as low as 45 Gy could fully sterilize the females of both strains and the males of the *w*Ha strain, whereas the *w*Tei males were 99% sterile. In addition, this low dose did not have any significant effect on longevity, adult emergence and flight ability. Mitotic and polytene chromosome analysis was also performed in the strain used in all SIT studies, an analysis which will be useful for the development and characterization of GSS for this species [82].

### 4.13. Lepidoptera

Lepidoptera are amongst the most severe pests of agricultural crops worldwide. Many control options exist, but they have issues of cost and efficiency. The SIT would be an additional control tactic that has great potential for the suppression or eradication of key Lepidoptera pests. Although there are no Lepidoptera colonies maintained at the IPCL, some research has been carried out with the codling moth *Cydia pomonella* and the date moth *Ectomyelois ceratoniae*.

In many instances, one strain of a pest is colonized and used in different geographical areas in the frame of SIT programs. In those cases, the absence of mating barriers between the released strain and the target strain is crucial for the success of the SIT. Mating compatibility studies in walk-in field cages were carried out with 12 codling moth populations originating from laboratories or from the wild from both hemispheres. In only two of the tested combinations was there indication of a deviation from random mating, indicating the absence of mating barriers between codling moth populations from many parts of the world [83].

The dose response effects of gamma radiation on the date moth was evaluated as a prerequisite for using the SIT/F_1_ against this pest. F_1_ sterility or inherited sterility (IS) is a derivative of the SIT and was developed for lepidopteran pests [84]. The irradiation dose that moths receive completely sterilizes irradiated female moths and partially sterilizes the males. Moths treated with this lower sterilizing dose live longer, are stronger fliers, and mate more frequently than moths treated with higher radiation doses. Moreover, the sterility effects are inherited by the offspring of the substerile males mated with wild fertile virgin females to produce completely sterile F_1_ moths—predominantly males [85]. A dose of 350 Gy was required to completely sterilize male date moths, whereas no eggs hatched from females irradiated with 300 Gy. Radiation induced effects in offspring from irradiated males and females indicated reduced fecundity, egg hatch, longevity and adult emergence over subsequent generations [86].

## 5. Main Research Achievements—Livestock Pests

### 5.1. Tsetse Rearing and Handling

Tsetse flies have a unique biology among insects, in that they reproduce by adenotrophic viviparity. Female tsetse flies produce one offspring every 10 days, which makes mass-production for the SIT a major challenge. Therefore, the IPCL has focused during the last decade on improving tsetse mass-rearing through a better understanding of the tsetse-symbionts-pathogens interactions that might affect its biology [87].

The IPCL has maintained colonies of tsetse flies according to the needs and requirements of FAO and IAEA member states. In the last 10 years, colonies of *Glossina palpalis gambiensis*, *Glossina fuscipes fuscipes*, *Glossina pallidipes*, *Glossina morsitans submorsitans*, *Glossina morsitans morsitans*, *Glossina morsitans centralis* and *Glossina brevipalpis* have been maintained, and biological material from these colonies have been provided to member states for a large array of studies. At the IPCL, the colonies are mainly utilized for inhouse experiments that investigate aspects such as colony maintenance [88] and establishment [89], tsetse symbionts and pathogens [87,88,90], sex separation [91], morphology [92], sterile male quality [93], radiation biology [94], mating behavior [95], transportation [96] and release systems [97].

Blood collection and processing factors such as anticoagulants, phagostimulation and blood source (from different hosts) may influence the quality of the blood meals for tsetse colonies. Defibrinated bovine blood was found to be suitable for feeding *G. brevipalpis* and *Glossina austeni*, but pupal production was increased by combining porcine blood with bovine blood or by adding the phagostimulants inosine tri-phosphate, cytosine mono-phosphate and guanosine mono-phosphate to the blood meal [98].

To optimize the yield of sterile *G. f. fuscipes* and *G. pallidipes* males without compromising colony productivity, a 1:4 male to female sex ratio in the production cages provided the highest fecundity combined with lowest mortality for both species [99].

As the SIT requires the sterile males to mate with wild females, a good understanding of the male reproductive system is therefore important. Techniques to observe *G. pallidipes* male genital were developed [92] and the composition of the spermatophore in *G. morsitans morsitans* was determined [100].

Tsetse flies reproduce very slowly, and females must be retained as much as possible in the rearing facility to produce offspring. Therefore, the ability to distinguish between males and female pupae will be a great advantage, because pupae are easier to transport, more robust to handle and larger volumes can be irradiated simultaneously. Near InfraRed (NIR) photography and video was used to observe and record intrapuparial development and the method could distinguish male from female *G. p. gambiensis* about five days before emergence [91], potentially allowing the male pupae to be handled and sterilized separately while leaving the females intact for the colony maintenance. The wings of the female melanize two days before the males and this sex difference was used to develop a Near Infrared Pupal Sex Sorter (NIRPSS) that is currently being tested [101].

The sterile males that are released for SIT are routinely marked with fluorescent dye powders to differentiate them from their wild counterparts and to estimate sterile to wild male ratios. Fluorescent dye powders are not always reliable, and therefore, alternative marking techniques have been investigated. Labelling colony *G. pallidipes* males with different stable isotopes indicated that they could be discriminated from wild flies with 95% certainty up to 85 days after release [102]. A molecular technique based on the determination of cytochrome oxidase haplotypes of *G. p. gambiensis* indicated that colony flies reared at the Centre International de Recherche-Développement sur l’Elevage en Zone Subhumide (CIRDES) shared the same haplotype in the 5′ end of the mitochondrial gene cytochrome oxidase I, which made them 100% distinguishable from wild flies collected from Senegal and Burkina Faso [103].

### 5.2. Mating Compatibility and Competitiveness

Assessing tsetse mating behavior under field conditions is challenging and expensive, and the results can be influenced by several environmental, climatic and ecological parameters which cannot be controlled. Walk-in field cages have been utilized successfully as a surrogate for field studies to conduct mating compatibility, mating competitiveness and other behavioral studies with fruit flies [104], lepidoptera [105], mosquitos [106] and tsetse flies [89].

**Figure 3:**
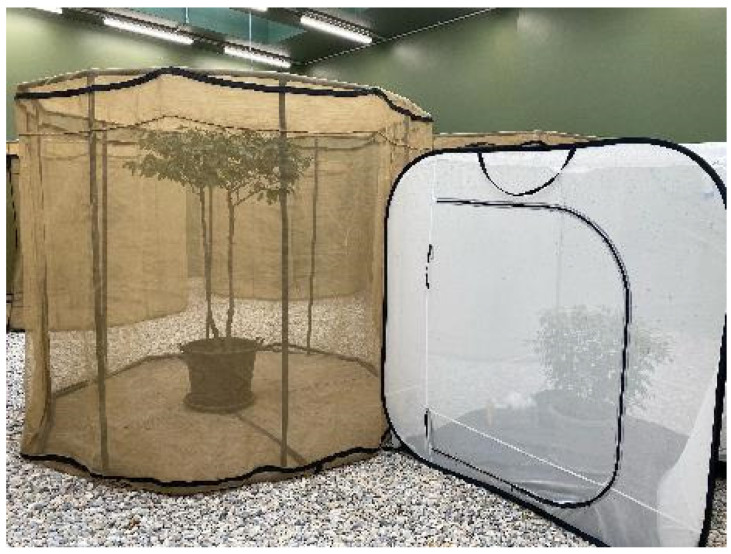
Walk in field cage for insect sexual behavior studies. (Photocredit: C. de Beer).

The use of sterile male flies that originate from one country and that will be used for release in other countries requires mating compatibility and competitiveness studies. Such an exercise was carried out in West Africa, where a laboratory colony of *G. p. gambiensis* was maintained at the CIRDES [89]. This Burkina Faso (BKF) strain was colonized in 1975 from seed material collected in Mare aux Hippopotames, Burkina Faso. To assess whether this BKF strain can be used for SIT in target areas of Mali and Senegal, colonies of local strains were established. Walk-in field cage (Figure 3) mating performance assessments of the three *G. p. gambiensis* strains indicated that mating barriers were absent and that the BKF males could be used for releases in Mali and Senegal [89].

The optimal mating age for *G. brevipalpis* and *G. austeni* was also determined in walk-in field cages. Age was identified as a factor that can influence mating competitiveness, and it was recommended that nine day-old or older males be used in the implementation of the SIT [107]. The effects of low temperature storage of irradiated *G. palpalis gambiensis* male pupae were also assessed. No direct impact on mating activity could be detected for male pupae stored at low temperature for periods up to seven days at the end of the male pupal period [94]. A new prototype of an automated chilled adult release system (Bruno Spreader Innovation, (BSI™)) for tsetse flies was tested. From the walk-in field cages evaluations, no significant negative effect on the male mating competitiveness could be observed, and the only negative effect was measurable in the survival rate [97]. The BSI™ release system has been identified as promising for use in future tsetse SIT programs.

A field evaluation of the mating competitiveness of a 40-year-old *G. p. gambiensis* colony indicated that the sterile males were able to induce nearly complete sterility in the wild female population when a sterile to wild male ratio of 10:1 was obtained [108]. Furthermore, a study on the performance of the BKF and Senegal (SEN) strains in an urban area of Senegal showed lower daily mortality rates for the SEN strain, but the BKF strain was more competitive [109].

### 5.3. Tsetse Symbionts

**Figure 4:**
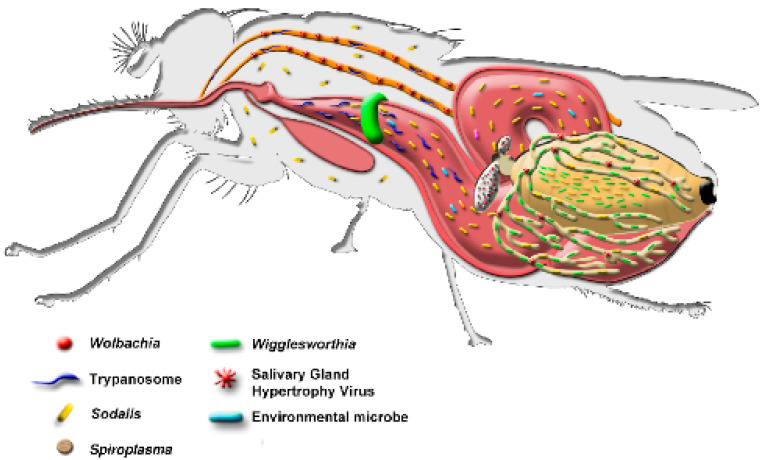
The tsetse fly and its associated microorganisms. Figure adapted with permission from [116,117].

Tsetse flies harbor several bacterial symbionts (gut microbiota and endosymbionts) that play an essential role in their biology (Figure 4), e.g., the endosymbiont *Wigglesworthia glossinidia* complements the tsetse diet by producing B vitamins [110]. In addition, *Sodalis glossinidius*, *Wolbachia* and *Spiroplasma* reside in tsetse flies [54,111], and although no clear beneficial role has been linked to these symbionts to date, *Sodalis* seems to enhance trypanosome infection, and *Wolbachia* has been shown to affect many aspects of the biology of its hosts, including host reproduction, development, immunity and behavior [112,113]. Knowledge of tsetse symbiosis is important to understand the fecundity, nutrition and immunity of the flies in order to develop novel approaches for their control [114,115].

To understand the role of endosymbionts and pathogens in tsetse flies, the IPCL has been the main driver of studies to assess the prevalence of endosymbionts (*Wolbachia* and *Sodalis*), salivary gland hypertrophy virus (SGHV) and trypanosomes in natural tsetse populations. The prevalence of *Wolbachia* was assessed in nine tsetse species collected in 10 countries. In addition, adults from six laboratory colonies were screened for *Wolbachia* infection. *Wolbachia* was most prevalent in *G. morsitans morsitans*, *G. morsitans centralis* and *G. austeni* populations. *Wolbachia* was also found in *G. brevipalpis*, and, for the first time, in *G. pallidipes* and *G. palpalis gambiensis* [118], but was not detected in *G. palpalis palpalis*, *G. fuscipes fuscipes* and *G. tachinoides* and only at a very low level (<1%) in *G. morsitans submorsitans*, and *G. medicorum* [118,119].

To improve the detection of low titer *Wolbachia* infections, the IPCL contributed to the development of sensitive detection tools using either the highly sensitive PCR-blot technique [120] or the high-end Stellaris^®^ rRNA-FISH technique [121]. The density of *Wolbachia* was high in tsetse hybrids which led to the assumption that *Wolbachia* might play a role in hybrid sterility of *G. morsitans centralis* males when mated with *G. morsitans morsitans* females.

The prevalence of SGHV was determined in natural tsetse populations [122] and its association with other tsetse symbionts and trypanosomes assessed [119]. The prevalence of the *G. pallidipes* SGHV (GpSGHV) in *G. pallidipes* varied between 2% and 100%, depending on the sampling location; however, phylogenetic and gene genealogy analyses revealed low virus diversity [122]. The prevalence of SGHV in other tsetse species in eastern and southern African countries indicated a lower prevalence in *G. morsiatns morsitans* from Tanzania (58%) and Zimbabwe (20%) than *G. pallidipes* collected in Tanzania (88%). The SGHV prevalence was low in *G. fuscipes fuscipes* (25–40%) from eastern Uganda [123,124] while the lowest prevalence (<2%) was found in species in West Africa such as *G. palpalis gambiensis*, *G. morsitans submorsitans*, *G. tachinoides* and *G. medicorum* [119].

Understanding the interaction between trypanosomes and tsetse symbionts is important to elucidate the vectorial capacity of the sterile males released in the frame of SIT programs. In addition, knowledge on trypanosome prevalence in tsetse flies might be used to predict the trypanosome prevalence in humans and livestock without the laborious and expensive parasitological studies. In this respect, the prevalence of trypanosome infection was very high in *G. tachinoides* (61.1%) from Ghana and in *G. palpalis gambiensis* (43.7%) from Senegal and very low in *G. palpalis gambiensis* from Mali (6.9%) and Guinea (2.2%). The trypanosome prevalence in the four species from Burkina Faso was 39.6% in *G. medicorum*, 18.1% in *G. morsitans submorsitans*, 16.8% in *G. tachinoides* and 10.5% in *G. palpalis gambiensis* [119]. Although coinfection of several trypanosome species was detected, the coinfection of trypanosomes with *Wolbachia* and SGHV was not found in this study [119] indicating a possible antagonistic effect or competition for host resources.

### 5.4. Tsetse Symbionts and Irradiation

The radiation dose used to sterilize male tsetse flies before release in SIT programs may potentially affect the associated microbiota which, in turn, might reduce fly performance. In addition, the tsetse-associated microbiota may influence their vectorial capacity; therefore, understanding the effects of irradiation on the symbionts is important, especially when sterile flies are released in areas endemic for human sleeping sickness [116,125,126]. Several studies reported the possibility of using modified *Sodalis* to produce antitrypanosome molecules within a paratransgenesis approach [127,128,129,130]. This might lead to the use of sterile male tsetse flies that are refractory to trypanosome transmission. To this end, irradiation with a dose normally used to sterilize tsetse males reduced the density of *Sodalis* when 29-day old pupae or adults were irradiated, but not when 22-day old pupae were irradiated. No significant impact of irradiation treatment was found on *Wigglesworthia* and *Wolbachia* densities. In addition, the vectorial capacity of the sterile males for trypanosome was not affected by an irradiation treatment [131].

### 5.5. Symbionts, Cuticular Hydrocarbons and Mating Choice

Recent studies suggest microbial involvement in chemical communication and mating behavior, which can ultimately impact reproductive isolation, and hence, speciation [132]. As mating behavior, reproduction and speciation are of paramount interest for the implementation of SIT programs. The IPCL participated in a study to investigate whether a disruption of the microbiota through antibiotic treatment (ampicillin, tetracycline) or irradiation affects cuticular hydrocarbon (CHC) profiles, and possibly mate choice behavior in the tsetse fly *G. morsitans morsitans.* The results indicated significant effects of antibiotic treatment (particularly tetracycline) on cuticular hydrocarbon profiles in both females and males, while irradiation itself had no obvious effect on the CHC profiles. In addition, tetracycline treatment reduced relative amounts of 15,19,23-trimethyl-heptatriacontane, a known compound of the female contact sex pheromone, suggesting a possible implication of microbiota disturbance on mate choice decisions and therefore both female and male flies preferred nontreated over tetracycline-treated flies in direct choice assays [133].

### 5.6. Spiroplasma

Until recently, it was believed that *Wigglesworthia*, *Sodalis* and *Wolbachia* were the only symbiotic bacteria present in tsetse flies. This notion was recently challenged with the discovery of the presence of two strains of another symbiotic bacterium, *Spiroplasma*, in both field and laboratory populations of *G. fuscipes fuscipes* and *G. tachinoides* [54]. The role of *Spiroplasma* is currently under investigation; however, it is important to note that the infection levels detected in *G. fuscipes fuscipes* were higher in larval than adult guts, in testes than in ovaries, and in live females than in those which died prematurely [54].

The presence of other bacterial species was assessed in three laboratory colonies of *G. p. gambiensis* using 16S *rRNA* gene-based next generation sequencing approaches. The analysis showed that there is clear tissue tropism resulting in different bacterial profiles between the gut and the reproductive organs [55]. The structure of the bacterial communities was similar among the three colonies with higher diversity observed in larval than in adult guts while no difference was observed between testes and ovaries profiles [55]. As expected, *Wigglesworthia* and *Sodalis* were the most abundant species; however, *Wolbachia*, *Propionibacterium* and *Providencia* were also detected [55].

### 5.7. Salivary Gland Hypertrophy Virus (SGHV)

In nature, tsetse flies exist at very low densities, but in tsetse mass-rearing facilities, the adults are kept in cages at relatively high density. Blood feeding is done using preheated defibrinated blood using an in vitro membrane feeding system that receives a successive number of fly-holding cages. These artificial conditions differ significantly from the natural environment and put additional stress on the flies that might affect their immune system, making them more vulnerable to pathogen infections. This might result in reduced productivity, increased abortion rates, and a higher percentage of small-sized pupae and, consequently, smaller adults. The worst-case scenario is the collapse of the colony, as was experienced with *G. pallidipes* at the IPCL in 2002. The problem proved to be associated with the presence of the *G. pallidipes* salivary gland hypertrophy virus (GpSGHV; hereafter referred to as SGHV).

**Figure 5:**
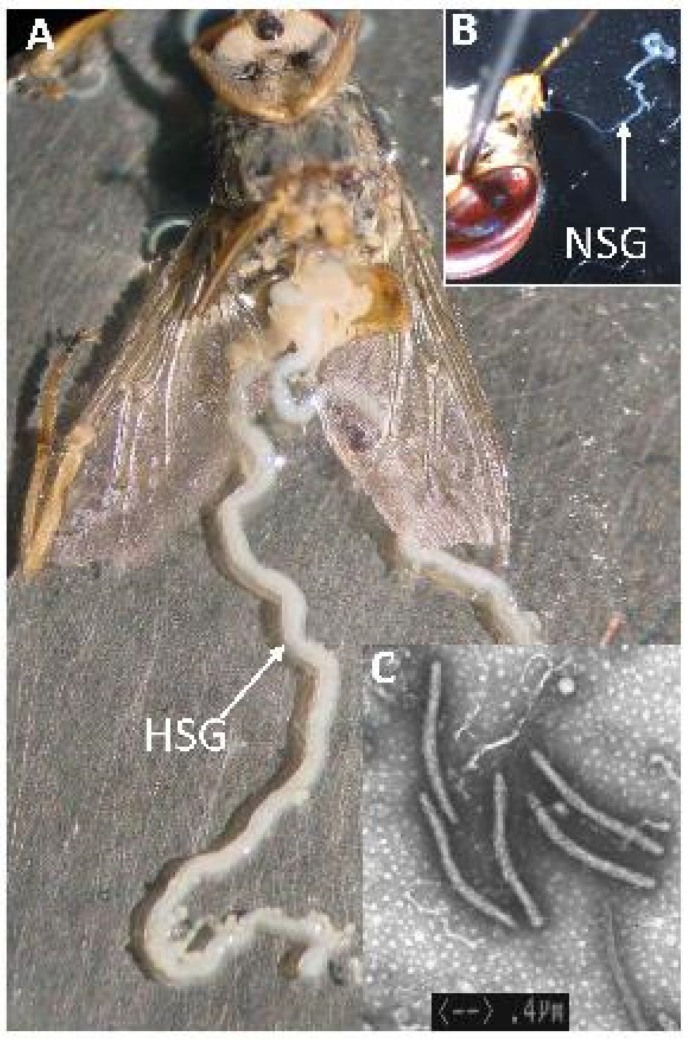
(**A**) Dissected adult *Glossina pallidipes* showing Hypertrophy Salivary Gland (HSG) symptoms caused by the *Glossina pallidipes* salivary gland hypertrophy virus (GpSGHV), (**B**) Normal salivary gland (BGS) relative to the adults tsetse head, (**C**) Transmission electron microscopy (TEM) micrograph of GpSGHV virus particles.

Symptoms of salivary gland hypertrophy (SGH) in wild tsetse flies (Figure 5) were first observed in 1932 [134,135,136], and in 1978, these were associated with virus particles [137]. Flies with SGH symptoms were found in several tsetse species that showed abnormalities in their reproductive organs resulting in reduced fecundity [138,139,140,141,142,143,144]. However, no problem was reported in colonized tsetse flies at that time.

Following the successful eradication of a population of *G. austeni* from Unguja Island of Zanzibar, United Republic of Tanzania [145], the Government of Ethiopia embarked on a project to use the SIT to eradicate a population of *G. pallidipes* and *G. fuscipes fuscipes* in the Southern Rift Valley. To establish a seed colony, 500 *G. pallidipes* pupae were collected from trapped wild females and shipped to the IPCL. The colony was expanded to 15,000 females in 2001. Thereafter, the colony started declining until it collapsed at the end of 2002. Dissections of colony flies indicated that around 80% of them showed SGH, which seemed to be associated with this unexpected demise of the colony. However, no such high prevalence of SGH symptoms was ever noticed in a second *G. pallidipes* colony that originated from Tororo, Uganda and that has been maintained in the IPCL since 1975.

A research program was started at the IPCL with the aim of developing a strategy to control the virus infection in *G. pallidipes* colonies. A study to better understand the dynamics of the virus infection showed that *G. pallidipes* flies with SGH symptoms release around 10 million virus particles in the blood during feeding in the laboratory. These virus particles, released in the blood, infect healthy flies that feed on the same blood. Asymptomatic flies (no symptoms of SGH) had no loss of productivity, but symptomatic male and female *G. pallidipes* (showing SGH symptoms) were almost completely sterile (males) or lost 50% of their productivity (females). In addition, offspring of females with SGH had SGH symptoms [146] and male *G. pallidipes* with SGH symptoms were significantly less competitive for mating with females in walk-in field cage studies compared with noninfected males [95]. SGHV can infect other tsetse species without developing SGH symptoms which can reduce productivity and survival of the flies, e.g., *G. fuscipes fuscipes* [147,148].

These studies revealed the important role of horizontal virus transmission in tsetse colonies through multiple feeding on the same membrane, and provided guidance for the development of a virus management strategy. It also explained the low prevalence of the virus in natural tsetse populations where transmission is mainly vertical from mother to offspring [90].

### 5.8. Classification of the Salivary Gland Hypertrophy Virus

To determine the relationship between the SGHV with other large DNA viruses, the genome of the SGHV from *G. pallidipes* populations from Ethiopia and Uganda was sequenced [149,150] and compared with other DNA viruses. The isolates from the Ethiopia and Uganda populations were two strains of the same virus, which was different from all DNA viruses and therefore, was classified in a new virus family called *Hytrosaviridae* [151,152,153,154,155].

SGH symptoms in *G. pallidipes* flies develop when cells of the salivary gland epithelium proliferate and form a multiple cell layer. In the house fly *Musca domestica*, however, infected flies with SGH symptoms develop an epithelium of the salivary gland that is composed of one layer of enlarged cells. Therefore, both viruses GpSGHV and mdSGHV were classified in the family of Hytrosaviridae but in two different genera, i.e., Glossinavirus and Muscavirus [155,156]. In addition, the structure of the SGHV virus seemed to be fragile [157,158,159,160], and a proteomic analysis revealed two major proteins in the virion that might be used to induce immune intervention against the virus infection [161]. Moreover, a SGHV infection altered the protein expression in the host [162]. To understand the nature of symptomatic infection in *G. pallidipes* and the asymptomatic infection dominant in other tsetse species, i.e., *G. m. morsitans*, a proteomic analysis in both species infected with the virus revealed distinctive differences in the protein expression profile that might explain the different infection status in both species [163,164]. In addition, a trans-generation transmission study revealed that injecting the SGHV in adults increased the virus titer but did not induce the development of SGH symptoms. These were however detected in the F_1_ offspring of injected flies. This might indicate that transmission of the SGHV in infected flies to its offspring most probably relies on the presence of one of the tsetse endosymbionts, probably *Sodalis*, as the treatment of the flies with an antibiotic blocked virus transmission to the F_1_ progeny [165].

During a study using microRNA and RNA interference, virus genetic diversity in natural tsetse populations was analyzed, revealing that the SGHV encodes for six microRNAs that may not only target the host immune system, but may also participate in the viral immune evasion; therefore, these microRNAs could be considered as an antiviral treatment [166,167]. The genetic diversity study of the SGHV revealed that the strain detected in *G. pallidipes* from Tororo, Uganda was the most prevalent strain. Therefore, it appears that *G. pallidipes* is the most recent tsetse species to be infected with this virus, and it can be speculated that this species is not yet well adapted to a virus infection, which might explain the high prevalence of SGH symptoms and, in certain cases, the collapse of these colonies [123,168].

### 5.9. Antiviral Drugs

A virus management strategy for tsetse fly colonies could have two objectives: (1) to prevent healthy flies from becoming infected with the virus; and (2) to delay or block virus replication to prevent an increase in virus titer to the level that causes SGH symptoms. Developing a management strategy required a better understanding of how SGH symptoms develop and why some flies have asymptomatic infection [90].

To mitigate virus replication during the early stages of infection, the potential of using available antiviral drugs against large DNA viruses, i.e., herpesviruses, was investigated. The screening of 15 antiviral drugs for their toxicity for tsetse flies indicated promising results for acyclovir and valacyclovir. The continuous use of valacyclovir at 300 µg/mL of blood was effective in reducing the virus titer without having a negative impact on the flies’ performance [169]. The drugs could be easily mixed with the blood meals and this method was recommended as an important part of the strategy to manage the virus in *G. pallidipes* colonies (as was done for the National Institute for Control and Eradication of Tsetse and Trypanosomosis (NICETT) facility in Kaliti, Ethiopia). Although the valacyclovir treatment was effective in protecting healthy flies or flies with a low virus titer during the early stages of an infection, it was not effective for flies with a high virus titer during the late stages of infection [169,170].

### 5.10. Clean Feeding Protocol

Another strategy was developed to control virus infections in tsetse colonies and prevent horizontal transmission based on improved colony management. Horizontal transmission of the virus can be avoided by feeding each batch of flies on a new lot of “clean” blood. This, however, is from a colony management point of view, challenging as it will increase the cost, as more materials (membranes and blood), more space and manpower will be needed, making the SIT less cost-effective. However, an alternative method to implement a clean feeding system without an increase of existing resources by dividing the colonies and changing the management recording system was developed. The details of this system are described by Abd-Alla et al. [88]. This study clearly indicated the possibility to eliminate the SGH symptoms from an infected colony within 28 months after the start of implementing this approach [88]. Although this study proved for the first time the feasibility of eliminating SGHV from a colony, the relatively long period required to do so was considered a drawback. Combining the clean feeding system with the use of antiviral drugs could eliminate SGH symptoms from a colony with a SGH prevalence of 24% within six months [170]. This approach was implemented for the *G. pallidipes* colony in NICETT and resulted in a stable colony with a sustainable output in sterile males for the implementation of the SIT program [171,172].

### 5.11. Tsetse Genomics and Wolbachia Infections

The IPCL participated in the “International Glossina Genome Initiative”, an effort that resulted in the sequence, assembly and annotation of *G. morsitans morsitans,* a major tsetse fly species, setting the ground for the harnessing of the genome data for the control of the vector and the disease (HAT and AAT—human and animal African trypanosomosis) [173]. One of the most interesting discoveries during the analysis of the genome was the multiple integrations of *Wolbachia* genome sequences in the tsetse chromosomes, including the two sex chromosomes, X and Y, and the supernumerary B chromosomes. These chromosomal insertions are maintained together with an active cytoplasmic *Wolbachia* infection, raising important questions about their impact on insect host biology, including vectorial capacity, evolution of tsetse species as well as on pest and disease control [173,174].

Recently, the genome sequence of additional five tsetse species was completed, i.e., *G. austeni*, *G. brevipalpis*, *G. fuscipes fuscipes*, *G. pallidipes* and *G. palpalis gambiensis*, which differ in their ecological habitats, target host species and vector competence, thus revealing more information about the unique biology and ecology of *Glossina* species in support of existing or novel pest and disease control strategies [175]. Interestingly, *Wolbachia* genome sequences were also found in the genome of *G. austeni*, which also contains an active cytoplasmic *Wolbachia* infection [175]. It is worth noting that the presence of chromosomal and cytoplasmic *Wolbachia* gene sequences together with a nuclear marker (internal transcribed spacer 1, ITS1) allowed us to develop a quick, effective and robust protocol for the accurate identification of several tsetse species and subspecies, irrespectively of their origin (field, laboratory or museum specimens) http://www-naweb.iaea.org/nafa/ipc/public/SOP-for-tsetse-species-identification-Final_8.pdf (accessed on 10 February 2021) [176].

### 5.12. Sterile Male Quality and Irradiation

The successful implementation of SIT depends on several requirements [177], of which the biological quality and sexual competitiveness of the sterile males are amongst the most important [1]. The sterile males must be able to compete with wild males for mating opportunities with the local virgin females [178], and hence, quality management assessments have been an important aspect of the IPCL research. A flight test has thus been developed and tested to monitor the quality of sterile male *G. palpalis gambiensis* throughout the production and transport processes to support the tsetse eradication program in Senegal [179,180].

In preparation for the SIT of *G. brevipalpis* and *G. austeni* the radiation sensitivity of colonized flies treated either as adults or late-stage pupae was determined. A dose of 40 to 80 Gy induced 97–99% sterility in *G. brevipalpis* females when mated with males irradiated as late-stage pupae or adults [181]. Higher doses, 80 to 100 Gy, were required for *G. austeni* males irradiated as late-stage pupae or adults to induce similar levels of sterility in females [182]. The mating performance of colony flies irradiated as adults and late-stage pupae, as investigated in walk-in field cages, indicate the irradiated males to be as competitive to mate with females as un-irradiated males [181,182].

### 5.13. Research in Direct Support of Tsetse AW-IPM Programs

The IPCL has long been contributing to feasibility studies for the development of AW-IPM strategies. Some of these studies were related to the collection of entomological, veterinary, population genetics, environmental and socio-economic baseline data.

A stratified entomological sampling scheme, using spatial and mathematical modeling, was developed [183] and used to determine the size of the target area in the AW-IPM program that aimed at eliminating a *G. p. gambiensis* population from the Niayes of Senegal. The parasitological and serological prevalence of *Trypanosoma congolense* and *Trypanosoma vivax*, the causative agents of African Animal Trypanosomosis (AAT), was assessed and was three times higher in the tsetse-infested than in the assumed tsetse-free areas [184]. Genetic differentiation between *G. p. gambiensis* from the Niayes and from the southern tsetse belt, Missira, indicated limited gene flow between these populations, providing evidence for the isolated character of the population [185]. This prompted the government of Senegal to select an eradication strategy [186]. The campaign was optimized by developing distribution models that investigated the relationships between tsetse presence and various remote sensing environmental parameters [187]. An ex-ante benefit-cost analysis predicted Internal Rates of Return (IRR) of 9.8% to 19.1% and a payback period of 13 to 18 years [188]. During the implementation of the program, the health of the ecosystem was monitored for nine consecutive years, using a set of fruit-feeding insect species (Cetoniinae and Nymphalidae) as ecological indicators. The suppression phase that involved the use of insecticides, reduced the apparent densities of the ecological indicators, but when the release of the sterile male insects started the apparent densities of the ecological indicators reverted to presuppression levels [189].

In the Senegal SIT program *G. p. gambiensis* is mass-reared in three remote production centers, i.e., the Centre International de Recherche-Développement sur l’Elevage en zone Subhumide (CIRDES) and the Insectarium de Bobo Dioulasso (IBD), in Bobo-Dioulasso, Burkina Faso and the Slovak Academy of Sciences (SAS), Bratislava, Slovakia. The supply of sterile male pupae is supplemented with male pupae from the IPCL. Irradiated male pupae were transported by courier service to the Institut Sénégalais de Recherche Agricoles, Laboratoire National d’Elevage et de Recherches Vétérinaires, Service Bio-Ecologies et de Pathologies Parasitaires (ISRA/LNERV/BEPP) in Dakar, Senegal [179,190]. The pupae were irradiated under chilled conditions with a dose of 110 Gy at the rearing insectaries in either Austria or Burkina Faso and transported under chilled conditions to the insectary in Senegal where they emerged after arrival and prepared for release. During long-distance transport, the chilling period and transport conditions were the main factors that influenced the quality of the pupae. The quality of the males can be improved by reducing the transport time and vibrations during transport as well as reducing or eliminating the time the pupae are chilled before the transport [96,180].

The environmental survival thresholds of the currently available three strains of *G. p. gambiensis* (Burkina Faso (BKF), Senegal (SEN) and a BKF/SEN hybrid were matched to a particular environment or ecosystem [191]. Survival and pupae production were more affected by temperature than by relative humidity, and the BKF strain was more resilient to high temperatures followed by the hybrid and SEN strains. The temperature limit of survival was 32 °C for all strains [191].

A new automated chilled adult tsetse release system was characterized in terms of the system’s ability to count the sterile males loaded into the machine, the consistency of the release rates and the impact on the quality of the released males. Sterile males that passed through the machine were less competitive as control males and this impact can be minimized by reducing the chilling duration [97].

In other West African countries, the structure of *G. p. gambiensis* and *G. tachinoides* populations was assessed using genetic markers to determine potential gene flow in four adjacent river basins in Burkina Faso, i.e., Mouhoun, Comoé, Niger and Sissili [192,193]. No strong barriers in gene flow were detected between riverine tsetse populations in the adjacent river basins [193]. This will need to be taken into consideration in the development of an AW-IPM strategy for Burkina Faso. As genetic analysis provides only indirect indications of tsetse movement between river basins, a release–recapture study was carried out that released sterile male *G. p. gambiensis* in tributaries of two river basins (Senegal and Bani) in Mali. This study clearly indicated that *G. p. gambiensis* can disperse between river basins in Mali, confirming the population genetics data [194].

As part of preparatory studies for the proposed AW-IPM strategy with an SIT component in Southern Africa, the distribution of the target species *G. austeni* and *G. brevipalpis* was determined [195]. The updated distribution maps showed that the South Africa populations extend into southern Mozambique and eSwatini (former Swaziland). Molecular and morphometrical markers were used to assess the degree of genetic isolation between these seemingly fragmented populations, and the data confirmed the absence of barriers to gene flow between the tsetse population from South Africa and southern Mozambique and limited gene flow between the eSwatini *G. austeni* populations with the South African and Mozambique populations [196].

Spatial models and distribution atlases [197] were developed to select suitable control tactics for a particular target zone and to predict the outcome of control interventions. These models focused on control tactics for tsetse eradication [198,199], tsetse distribution [200], habitat suitability [201,202], tsetse dispersal [203,204] and trypanosomosis risk [205].

An IPM campaign in Burkina Faso aimed to eliminate tsetse from an area of 40,000 km^2^ by integrating insecticide-treated traps, targets and cattle, the sequential aerosol technique (SAT) and mass-treatment of livestock using trypanocides. A monitoring exercise indicated that although tsetse densities were efficiently suppressed, the SIT may need to be integrated into the control campaign for the total elimination of tsetse from the area [206].

## 6. Main Research Achievements—Human Disease Vectors

### 6.1. Combining SIT and IIT for Mosquito Population Suppression

One of the major challenges for using SIT or any other genetic control method for the population suppression of mosquito vector species is sex separation, because released females, even if they are sterile, may still bite, blood-feed and transmit pathogens [207,208,209]. In the absence of an efficient and robust sexing system, the combination of SIT and IIT was proposed as a safe approach to suppress populations of *Aedes* mosquito species [210,211]. As mentioned above, in the combined SIT/IIT approach, females are fully sterilized by a low irradiation dose while males are sterile due to *Wolbachia* infection and the low irradiation dose. The advantage of this approach is that males are more competitive and that accidentally released female mosquitoes will be fully sterile and will have a reduced potential of transmitting pathogens such as dengue, chikungunya, Zika and yellow fever, due to pathogen interference phenomena mediated by the *Wolbachia* infection [210,211]. The combined SIT/IIT was successfully tested at the IPCL under laboratory and semifield conditions using the Asian tiger mosquito, *Ae. albopictus* as target species [212,213,214]. A new *Wolbachia* strain was transferred to a naturally double-infected *Ae. albopictus* strain and affected neither the rearing efficiency nor productivity or mating competitiveness of the male mosquitoes. In addition, the low irradiation dose fully sterilized the females, thus ensuring that the combined SIT/IIT strategy will not result in population replacement in the case of an erroneous release of fertile females [212,213,214,215]. The increase in production capacity of mosquito rearing facilities, such as the Wolbaki facility in Guangzhou, China, allowed the testing of this combined SIT/IIT approach under open field conditions in two sites in China [215]. The releases resulted in the successful suppression by more than 90% of the target *Ae. albopictus* population in both sites [215].

Mosquito strains used for population suppression approaches may need to be of local origin or introgressed into the local genomic background to address potential biosecurity or regulatory concerns. However, the introgression into a local genomic background may have an impact on the biological characteristics of the strain. This question was investigated using the yellow fever mosquito, *Ae. aegypti* carrying the *w*AlbB *Wolbachia* strain (WB2) which was introgressed into the genomic background of Brazil and Mexico populations. The results showed that the Brazilian genomic background had no significant effect on life-history traits while the introgression into the Mexican genomic background had a negative effect on fertility, longevity and pupal size [216].

### 6.2. Aedes aegypti Genetic Sexing Strains

The use of Mediterranean fruit fly GSSs have increased the effectiveness and cost-efficiency of the SIT. Using genetic approaches, two GSSs for the *Ae. aegypti* were developed based on eye color as a selectable marker, i.e., a red-eye GSS and a white-eye GSS [217]. In these GSSs, males have wild type black eyes while females have either red-eyes or white-eyes. Both the *red-eye* (*re*) and the *white-eye* (*w*) genes are located on chromosome 1, the chromosome which also carries the M locus that determines the male sex. Under laboratory conditions, the red-eye GSS had a better biological quality and genetic stability as compared with the white-eye GSS [217]. Red-eye GSS males, irradiated with 90 Gy and released in small laboratory cages at a 10:1 ratio to wild males, successfully suppressed a target laboratory population in six weeks [217].

Using irradiation, a chromosomal inversion (Inv35) was induced on the chromosome covering the M and *re* loci [218]. Inv35 significantly reduced the recombination rate between the M and *re* loci from about 2–3% to 0.2–0.3%, thus enhancing the genetic stability of the GSS [217,218]. In addition, an image analysis algorithm was developed which allows the discrimination of mosquitoes having black or red eyes at the pupal stage [217]. It should be noted, however, that the eye phenotype is evident throughout the development, from first instar larvae to adults. The combination of different biological traits and genetic tools, such as protandry, sexual size dimorphism at the pupal stage and the red-eye GSS with Inv35, may essentially eliminate the females and achieve male-only releases for SIT applications against *Ae. aegypti* [217]. The automation of this system is the next and crucial R&D step.

In the absence of a good sexing system, the separation of males and females *Aedes* sp. has been routinely performed based on the sexual size dimorphism of the pupae using sieving plates or the Fay-Morlan glass plate separator [219]. However, factors such as the day of pupae collection and the sorting operator affected the efficiency of the Fay–Morlan glass plate separators [220]. Automation of the sex sorter is being assessed to standardize the process and make it less variable. Gunathilaka et al. [219] suggested that spiking the blood with 8 ppm of ivermectin after applying the Fay-Morlan glass plate method, could achieve 100% separation of sexes with significantly lower amounts of toxicants. The contamination of the adult cages with insecticide residuals remains however, a serious drawback.

### 6.3. Anopheles arabiensis Genetic Sexing Strain ANO IPCL1

A first GSS of *An. arabiensis*, an important vector of malaria, was based on resistance to the insecticide dieldrin [221] whereby dieldrin-resistant males were exposed to low-dose gamma-ray irradiation and were then crossed to homozygous susceptible virgin females. The resulting strain was named ANO IPCL1, and was characterized and evaluated to assess its suitability for use in SIT programs [222,223]. The ANO IPCL1 strain was compared with the two wild strains from which it originated, and showed similar developmental parameters. In addition, the GSS had several advantages, i.e., the removal of females could be achieved at the egg or larval stage by exposure to dieldrin solutions, lower irradiation doses were required to obtain full sterility in this strain due its high natural sterility, and competitiveness of sterile males was similar to their fertile counterparts [223].

The presence of the Y-autosome translocation (which confers the resistance to dieldrin) affected male fertility resulting in only 27% egg hatch [222]. This issue, together with its 0.4% recombination rate (resistant females), would increase production cost significantly, and pose a risk of strain deterioration, requiring additional amplification steps and a filter colony in a mass-rearing. The main disadvantage of the ANO IPCL1 strain, however, that lead to a recommendation to not use it in operational programs, was the discovery that dieldrin-treated male mosquitoes retained residues of this toxic insecticide and could contribute to the bioaccumulation of these compounds in natural predators of the released mosquitoes, and the environment [224]. This fact, in addition to adverse health implications for mass-rearing staff chronically exposed to copious amounts of dieldrin, led to the investigation into alternative sexing methods for *An. arabiensis*.

More recently, an *An. arabiensis* temperature sensitive lethal strain was developed and evaluated in terms of its potential use for further development into a novel GSS [225]. The strain showed no major differences in life history traits compared to the wild type strain.

Like with *Aedes* mosquitoes, the requirement of a blood meal for females can be exploited for sex separation and early publications reported on mixing malathion in blood meals to kill adult female *An. albimanus* in the SIT program in El Salvador [226]. Several toxicants for spiking blood meals were tested and ivermectin, a less toxic antihelminthic drug, proved to be efficient to remove female *An. arabiensis* with no detrimental effects to the males [227]. Although this method is less efficient than GSSs, it has the advantage that it can be applied to any local strain for use in small-scale sterile male release efforts.

### 6.4. Rearing Mosquito Larvae

The successful application of the SIT requires the ability to rear the target insect in numbers large enough to obtain adequate overflooding ratios in the field. However, an artificial rearing environment might impose selective pressures that can lead to changes in behavioral and physiological traits. The process of colonization accelerated the sexual maturation of male *An. arabiensis* with 42% and 96% successful matings with females 11 h and 17 h after emergence, respectively, compared with the 24 h which are generally required for the completion of sexual maturation [228].

Optimization of mass-rearing procedures requires the ability to store and quantify eggs and reliably assess egg hatch rate [229,230]. Eggs can be accurately quantified by a method based on weight before being dispensed in rearing trays [231]. A hatch rate greater than 80% of 2 week-old *Aedes* eggs can be obtained when using boiled-cooled osmosis water or with a hatching solution, i.e., osmosis water with 0.25 g nutrient broth and 0.05 g brewer’s yeast [232]. Studies on optimum *An. arabiensis* egg drying and storage methods indicated that eggs, collected from mass-rearing cages, can be air-dried for 4 h or dried using a suction device (a pump creating a vacuum to remove fluid) set at 1.8 m/s wind speed for 20 min. Dried eggs can be used the same day or stored in bulk at 20 °C for up to 6 days without any reduction in hatch rate and larval development [233]. Eggs collected on damp filter paper and sealed in plastic bags can be stored at ambient room temperature for up to 4 days [234]. Egg loss due to psocids of the genus *Liposcelis* (Psocoptera: Liposcelididae) that scavenge on mosquito eggs, needs to be prevented in rearing facilities [235].

The availability of artificial larval and adult diets is crucial for cost-effective mosquito rearing, and artificial diets play a critical role in mosquito quality. Efforts made to substitute the expensive Koi Floating Blend^®^ Fish food used routinely for colony maintenance resulted in several suitable larval diets, i.e., a diet with bovine liver powder, tuna meal and Vitamix as ingredients for rearing *An. arabiensis* larvae [236], and a mix of bovine liver powder, tuna meal and brewer’s yeast for rearing *Aedes* larvae [237]. Larval diet quality and quantity (g/larva) influence adult quality and sexual competitiveness and therefore, these diets were tested for a wide range of larval densities and diet amounts on a laboratory scale for *An. arabiensis* [236,238,239], *An. gambiae* [240] and *An. coluzzii* [241] and at mass-rearing scale for *An. arabiensis* [242]. Bovine liver power is the most important ingredient in these diets but also the most expensive and it is not always and everywhere available. Therefore, cheaper alternatives based on grinded insects were tested for their nutritional value, i.e., meals of the yellow mealworm, *Tenebrio molitor*, the house fly *Musca domestica* and black soldier fly (BSF), *Hermetia illucens* were found to be sustainable alternatives [243]. Powder of BSF larvae proved to be the most promising with similar production and male flight ability under mass-rearing conditions and provided economic savings of at least 80% as compared to the reference diet [244]. Other cheap, locally available diets were tested by IAEA Collaborating Centers or through collaborative research projects, e.g., the laboratory rodent diet which performed well in terms of pupae production, adult longevity and male flight ability for the rearing of *Ae. aegypti* [245] and cat food, brewer’s yeast and tetramin fish food for the rearing of *Ae. albopictus* with no difference in male body size and female fecundity compared to the IAEA diet [237].

To assess the impact of dietary quality on subsequent adult quality of *An. arabiensis,* a technique was used based on elemental analysis and isotopes ratio using pyrolysis gas chromatograph/mass spectrometry. Dietary carbon and nitrogen have been shown to control teneral mosquito size. Therefore, total body carbon and total body nitrogen can be used as a measure of mosquito body size. Moreover, dietary phosphorus appeared to have a greater impact on adult production [246].

The maximum and optimal rearing densities for *Anopheles* spp. and *Aedes* spp. have been found to be one first instar larvae (L1) /mL and 3.6 L1/mL respectively [232,247] and thus rearing large numbers for larger SIT programs infers the necessity of numerous rearing trays and copious amounts of water, and thus large space requirements. To address these challenges with respect to space optimization and continued stability of larval rearing, a “rack system” was developed to accommodate up to 50 large, stacked rearing trays [248], each with a capacity to rear 4000 *Anopheles* larvae, or 18,000 *Aedes* larvae, within 1 m^2^ of floorspace. The system was successfully tested for *Ae. albopictus* [249], *Ae. aegypti* [220] and *An. arabiensis* [242]. Although some mass-rearing systems for mosquitoes, and other insects negatively affect adult biological quality, Soma et al. [250] demonstrated that the aforementioned mass-rearing system did not affect the longevity, body size and mating competitiveness of adult *An. arabiensis* mosquitoes compared to those reared at a small-scale. Cheaper versions (using aluminum rather than stainless steel) have been developed more recently, and other, similar rack systems have been derived from this rack model [251], in which the effects of different *Ae. albopictus* larval densities were tested and 2 L1/mL resulted in the highest male and pupal productivity [251,252].

Mass-rearing of immature stages of mosquitoes requires large volumes of water, and the quality thereof is critical for optimal larval development and the quality of the adults. In many areas of the world, osmosis water is a scarce or costly resource; therefore, the use of tap water for rearing *Aedes* mosquitoes was assessed. Tap water with hardness or electrical conductivity levels beyond 140 mg/L CaCO_3_ or 368 µS/cm was shown to be suboptimal, resulting in reduced productivity of *Aedes* colonies [253]. To minimize water usage and running costs, recycling larval rearing water via reverse osmosis and ultrafiltration processes to rear successive generations of *An. arabiensis* larvae was tested with good results [254,255].

Although the cost and automation are important factors for the implementation of large-scale SIT programs, the relation between those is not well established. To achieve consistent production and standardized rearing, the initial number of larvae present in each larval tray needs to be controlled. A custom-made automated counter for mosquito larvae was developed [256] that showed good accuracy and repeatability. The accurate counting of *Anopheles* and *Aedes* mosquito larvae will reduce manpower, increase time efficiency and have lower heterogeneity of larval density.

A cold-water vortex device (water temperature ranging 4–15 °C) was developed to separate larvae and pupae of *An. arabiensis* [257]. A mixture of 1 million larvae and pupae could be processed per hour using the difference in buoyant density and behavior between the larvae and the pupae.

### 6.5. Rearing Mosquito Adults

Adequate uptake of sugar and blood are important for the survival of adult mosquitoes and for egg production. A Hemotek blood feeding device was modified and shown to be suitable for blood feeding mosquitoes for several hours under mass-rearing conditions [258]. A new adult mass-production cage (200 (L) × 100 (H) × 20 (W) cm and 200 (L) × 100 (H) × 10 (W) cm for *Anopheles* spp. and 100 (L) × 100 (H) × 10 (W) cm for *Aedes* spp.) (referred to as the IAEA reference) with resting, mating, feeding, and oviposition sites was developed [259,260] and showed potential for the mass-production of *Ae. albopictus* [260]. Modifications such as an improved sugar-feeding device and addition of resting sites enhanced adult *An. arabiensis* survival and egg production [261]. The effects of cage volume, blood meal source and adult population density were assessed to optimize *An. arabiensis* egg production [262]. Rearing *Ae. aegypti* in a low-cost plexiglass mass-rearing cage (90 (L) × 90 (H) × 20 (W) cm) [263] showed similar egg production but better egg fertility as compared to the IAEA reference. The plexiglass mass-rearing cage was successfully tested in terms of egg production for *Ae. albopictus* and *An. arabiensis* [264].

Up to 10 million eggs per week can be produced with 23 *Aedes* mass-rearing cages stacked in a single layer in a 50 m² adult holding room. Collaborators from China developed a medium size mass-rearing facility with stainless-steel adult cages capable of sustaining a production of 10 million *Ae. albopictus* eggs within 15 days [265].

### 6.6. Irradiation

Inducing sterility in the wild female insect population is the underlying essence of the SIT. Reproductive sterility is obtained by breaking molecular bonds within the DNA of sperm cells, creating multiple dominant lethal mutations in the germ cells [266,267]. In addition to radiation dose-responses [268,269,270,271], work has focused on the effects of irradiation on mosquito fertility, longevity, flight ability and mating competitiveness [106,272,273,274,275,276,277]. Other factors such as handling, irradiation device and source, and intrinsic biological factors of the mosquito might also affect radiation-driven sterility and require research to enable the development of standard protocols for the reliable and reproducible induction of sterility [278].

**Figure 6:**
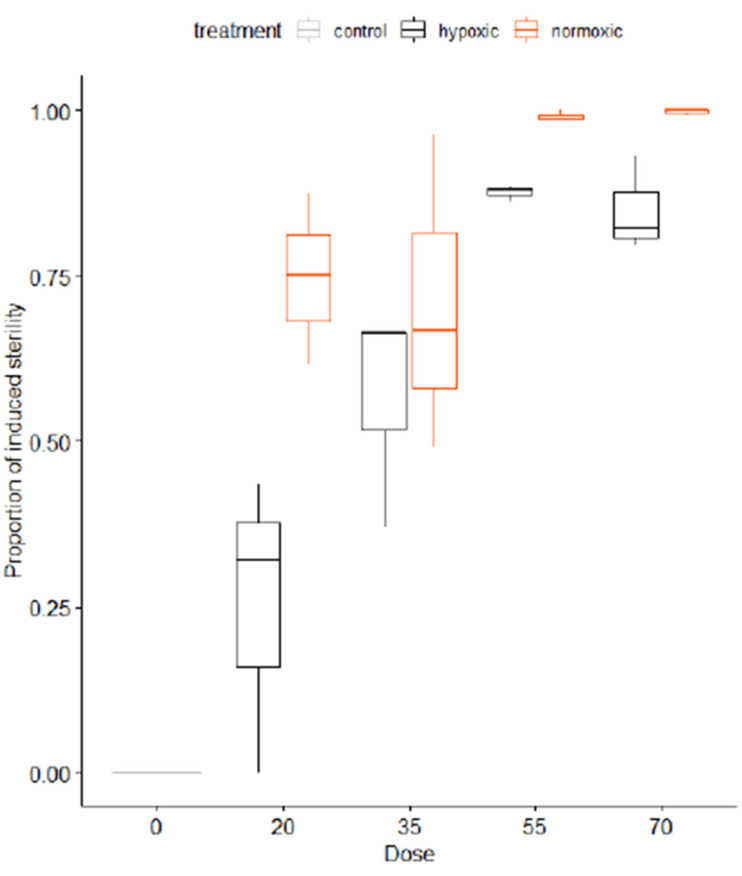
Effect of hypoxia during irradiation in *Aedes albopictus.* Hypoxia has significant protective effects, rendering irradiated males with lower sterility levels at all doses (*p* = 1.48 × 10^−5^) Figure from Yamada et al., 2020 [282].

Initial research focused on the radiation biology of *An. arabiensis* [279], and was later expanded to *Ae. albopictus* and *Ae. aegypti*. Both *Aedes* species required a lower dose to achieve full sterility than *An. arabiensis*, i.e., 40–60 Gy for >99% sterility in *Ae. albopictus* [280,281], 55–70 Gy for *Ae. aegypti* [282,283], and 100–120 Gy for *An. arabiensis* [268,284]. The study of Balestrino et al. [281] indicated that female *Ae. albopictus* (Rimini strain) pupae are more radio-sensitive than male pupae, and older pupae more resistant to radiation effects, corroborating similar studies in other insects. The longevity of *Ae. albopictus* in semifield cages was not affected by irradiation in the pupal stage, in the absence of females, but in the presence of females, the lifespan of fertile and sterile males was reduced. Providing sugar meals greatly improved longevity in this species and showed the importance of providing an energy source to sterile males prior to release [285]. Parameters such as pupal size and geographic origin did not have significant effects on sterility in both *Aedes* species, whereas handling methods and atmospheric conditions (absence of oxygen) during radiation exposure did greatly affect dose-response, by reducing “oxygen effects” [278], resulting in less sterility. The effects of hypoxia have been reported in several insect pests which were more resistant to the radiation treatment, with residual fertility in some cases increasing up to 20-fold versus those irradiated in air [286,287,288]. In addition, irradiation of *Ae. albopictus*, *Ae. aegypti* and *An. arabiensis* pupae in air or in water resulted in significant variation in induced sterility, indicating different oxy-regulatory behavior when submerged under water [282]. The effects were most pronounced in *An. arabiensis*, but all three species showed high protective effects (and thus reduced sterility) of hypoxia during irradiation (Figure 6).

A self-contained X-ray irradiator with maximum X-ray beam energy of 150 keV using a 4π X-ray tube was evaluated as an alternative to the traditionally used self-shielded gamma-ray irradiators [289] The dose rate at maximum power in the center of a canister filled with insects was determined as 14 Gy min-1 and the dose ratio to be about 1.3 [289]. An alanine-EPR dosimetry system was evaluated and found to be suitable as a dosimetry system [290]. X-ray machines and high energy linear accelerators have been used to sterilize mosquitoes, i.e., a dose of 40 Gy in *Ae. albopictus* [280] and *Ae. japonicus* (*Hulecoeteomyia japonica*) [291]. These results, among others have stimulated increased research into the application potential of X-ray technology for the sterilization of Aedine, as well as Anopheline [284] mosquitoes.

Insemination rates of field samples of *An. arabiensis* were higher compared with laboratory populations, and were overall lower in irradiated versus nonirradiated field and laboratory samples. Both irradiation and colonization altered reproductive traits and quality of the adults such as fecundity, egg hatch rates, larval viability and longevity, which were generally reduced [292]. Released irradiated, sterile male *An. arabiensis* were able to locate and participate in swarms in the field in Sudan. Although the survival probability decreased with the age of male when released, the distance travelled and swarm participation by older males seemed higher than for younger males [293]. These findings, together with previous reports on the importance of sugar meal provision [285], suggest the benefits of a prerelease period to enhance male competitiveness.

Irradiation affected vector competence of female *An. arabiensis* for the malaria parasite *Plasmodium falciparum*, with irradiation reducing the proportion of females infected with *P. falciparum* oocyst stages with 17%. However, the number of developing oocysts and the sporozoite infection rate and load was similar between irradiated and unirradiated females. These findings are relevant in case some females are accidentally released together with the sterile males [294].

### 6.7. Mosquito Symbionts

Similar to plant pests and livestock pests, studies were also carried out in *Aedes* mosquitoes to characterize the egg- and gut-associated microbiota in order to harness it for the enhancement of rearing efficiency and biological quality of strains for use in SIT applications. Using culture-dependent and culture-independent approaches, the egg- and gut-associated bacterial species in a laboratory colony of *Ae. albopictus* were characterized. The analysis was carried out with different developmental stages (larvae, adults), ages (young, old) and feeding conditions (sugar, blood). The data showed that the microbiota is diverse, dynamic and mainly affected by the developmental stage and diet [56]. Some of the detected bacterial species, mainly members of the *Enterobacteriaceae* and *Acetobacteriaceae*, are worth further investigation with respect to their potential probiotic properties.

### 6.8. Detection of Viruses in Mosquitoes

As the SIT requires the mass-rearing of mosquitoes in mass-rearing facilities, biosafety measures to ensure the safety of the staff with respect to avoiding infection from mosquito-borne viruses are crucial. A sensitive RT-qPCR detection method was developed to check and assess the infection level of newly sampled mosquitoes from the field. The method can both be used to assess the virus-free status of field collected samples and for routine checking of available colonies [295].

### 6.9. Mating Behavior

The amount of sperm in the testes of *An. arabiensis* and *Ae. aegypti* males increases with age [296,297], which ensures that females can be inseminated throughout a male’s life, even after successive matings. Irradiation decreased the initial amount of sperm in the *An. arabiensis* GSS strain ANO IPCL 1, and a subsequent loss of sperm over time with no new production thereof [298]. It remains unclear whether irradiated *Anopheline* males can recover their fertility, and this was not observed according to Patterson et al. [299]. Fertility was recovered in *Ae. albopictus* following irradiation with substerilizing doses [272].

Understanding the mating behavior of male mosquitoes in the field is essential for proper decision making. Anopheline mosquitoes mate in swarms, and the ability of irradiated males to find, initiate and participate in swarms is necessary for the success of the SIT for these species. Swarms of *An. arabiensis* were formed throughout the year and start and end times of the swarms varied according to the month correlating with the setting of the sun: swarms were formed at or after sunset from late July to early October, and before sunset the rest of the year [300]. This information can guide the timing of the releases to enhance the chances of sterile males joining the swarms. A study in Sudan confirmed that sterile *An. arabiensis* males were also able to participate and mate in swarms [293]. Biological factors that were found to affect mating success in males of an *An. gambiae s.s.* population in Burkina Faso included age and body size [301,302,303].

**Figure 7:**
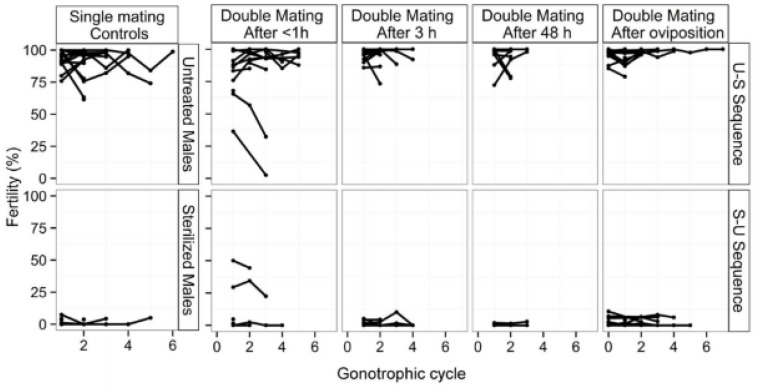
Fertility of female *Ae. albopictus* mated once with an untreated or sterilized male, or twice at various interval of time with males in untreated-sterilized or sterilized-untreated mating sequences. Individual fertility of females over multiple gonotrophic cycles [305].

The reproductive biology of (sterile) *Aedes* males differs greatly from that of Anopheline mosquitoes. Double-mating experiments with *Ae. albopictus* showed that multiple inseminations and thus potential sperm competition only occurred when consecutive matings were closely spaced time-wise [304], i.e., matings with two different males spaced longer than 40 min apart resulted in progeny with sperm provided by the first male (Figure 7). Sperm was replenished after a few days of rest in fertile males but not in sterile males. A sterile male could fully inseminate 7 females and partially inseminate another 8 females during its lifetime, compared with a fertile male that could fully inseminate 11 females and partially inseminate another 9 throughout its life [304]. However, similarly to a fertile male, a sterile male can transfer enough sperm to females to prevent further insemination (by for example, a fertile wild male), and is therefore expected to be equally reproductively competitive, providing that they survive and can locate females equally well [305].

The satyrization behavior of *Ae. aegypti* and *Ae. albopictus* that occurs sympatrically on Reunion Island has consequences for a management program against only one of them. In a study assessing sympatric, conspecific, interspecific and allopatric effects of sterile and fertile male *Ae. albopictus* on female *Ae. aegypti*, using varying male ratios, a low level of satyrization was observed between the two populations. This implies that the *Ae. aegypti* strain from La Réunion has developed resistance to satyrization; therefore, releasing sterile male *Ae. albopictus* will likely not suppress *Ae. aegypti* populations [306].

### 6.10. Quality Control

The competitiveness index *C* (also called Fried index) is a very commonly used quality control parameter in in SIT programs [307].

*C* values observed in pilot trials world-wide were, in general, higher than 0.2 [308], which is considered the lower limit for effective SIT projects. Any lower value would require an asymptomatic increase in the numbers of sterile males to be released [309,310]. Irradiation dose was negatively correlated with the *C* values, highlighting the importance to identify the optimal radiation dose through a dose-response curve but link it to competitiveness assessments for each particular strain, irradiator and environmental conditions [278]. *Anopheles* species required a higher dose to obtain the same level of sterility as compared with *Aedes* species. However, *C* was sometimes very variable at the same dose between experiments, e.g., a *C* value between 0.4 and 1 with a dose of 35 Gy for *Ae. albopictus*, showing that other factors than irradiation such as rearing conditions, handling, chilling, etc., are more important quality reducing. Irradiating adult *An. arabiensis* resulted in better insemination rates as compared with irradiating pupae, probably because irradiating adults induced lower levels of somatic damage than irradiating pupae which contain more stem cells. Moreover, contrary to adult irradiation, pupae irradiation can result in partial recovery of fertility with age [272]. The competitiveness index was higher for five-day old *Ae. albopictus* males as compared with one-day old males, which were not sexually mature [311].

**Figure 8:**
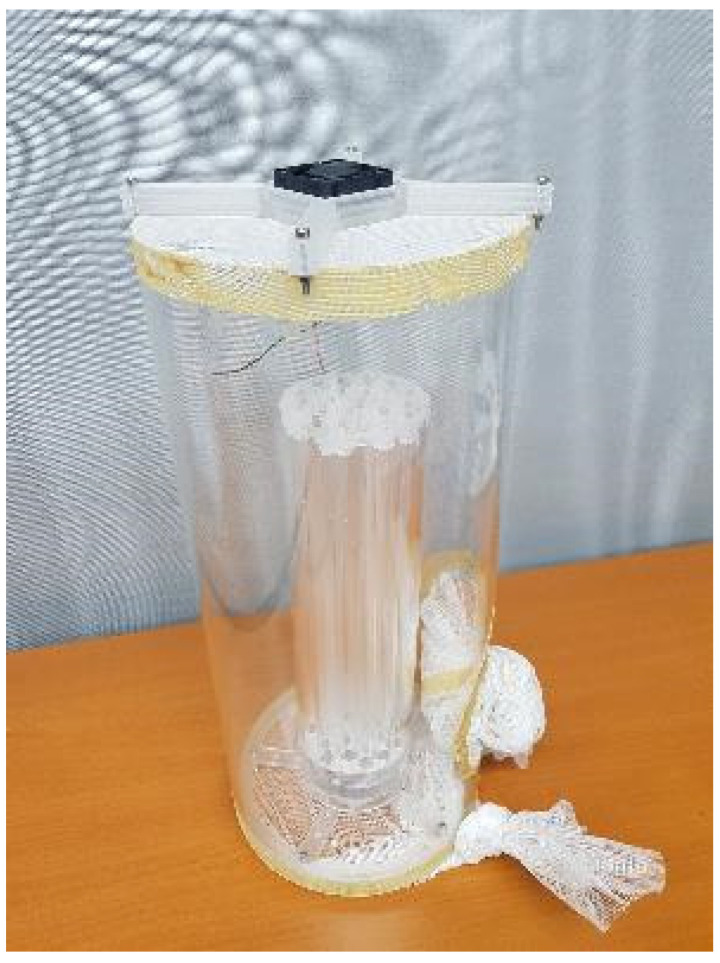
Flight ability test conducted on sterile male *Anopheles arabiensis* (Source: (Culbert et al. [314])) (Photo credit: H. Maiga).

A simple QC method was developed based on flight ability using flight cylinders [312]. This was further developed and proved to be a quick but accurate tool with which to evaluate the quality of sterile male mosquitoes; see Figure 8 [313,314]. A flight ability index corresponding to an escape rate within two hours was successfully tested against standard QC indicators such as survival and mating ability. Further research is needed to test this indicator against the *C* value measured in semifield or field conditions.

The environmental conditions for transport and release of sterile males were defined [315,316]. Temperatures between 8 and 12 °C were optimal for the transport of *Aedes* species, whereas a layer of adult mosquitoes of 5 cm thickness was the maximal compaction level preventing damage and reduced quality.

### 6.11. Research in Direct Support of Mosquito AW-IPM Programs

A method was developed for marking ~70,000 sterile chilled male mosquitoes in 20 min [317]. This approach was integrated into protocols to conduct mark-release-recapture experiments allowing the estimation of their survival, dispersal and competitiveness [318].

**Figure 9:**
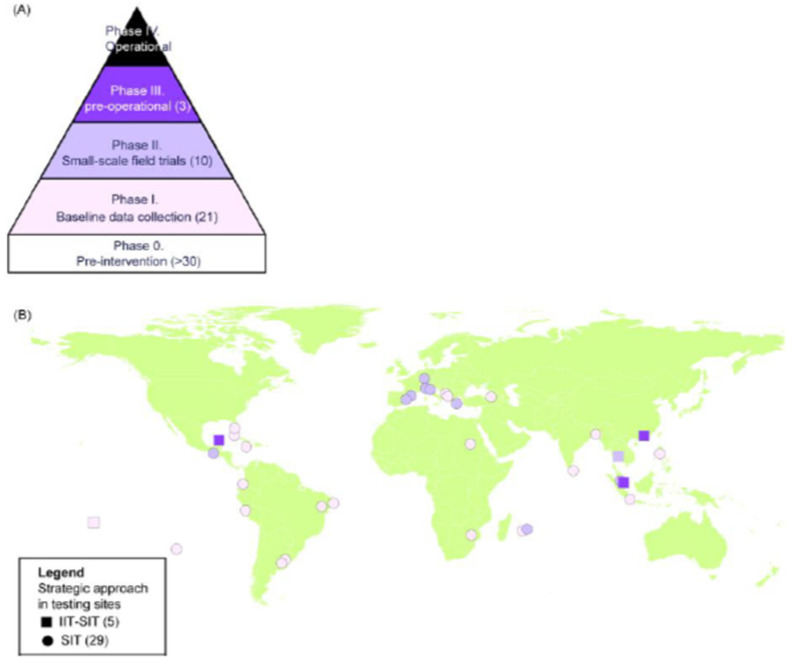
Schematic representation of the phased conditional approach (PCA) proposed to apply SIT and Location of the pilot sites in each phase. (**A**) The pyramid shows the amount of innovation related to operational research that is required in each phase, whereas the volume of activities and investment will generally grow in the opposite way. (**B**) Locations of field sites implementing the SIT against mosquitoes, some of which in combination with the incompatible insect technique (IIT-SIT). The number of field trials for each strategy are presented in brackets. Phase 0 sites are not included. (Source: modified from (Bouyer et al. [319])).

A phased conditional approach (PCA) for mosquito management using the SIT was proposed, where support to the next phase is conditional to the completion of activities in the previous phase and the scope, expense and commitment increase along the process [319]. The proposed phases are (0) pre-intervention, (I) baseline data collection, (II) small-scale field trial, (III) preoperational, and (IV) operational. Figure 9 presents a map of testing sites currently implementing SIT against mosquitoes, alone or in combination with the IIT.

At the time of writing, there are world-wide 13 trials where sterile mosquitoes are released, and an additional 10 where baseline data are collected [308]. Populations of *Ae. albopictus* were successfully suppressed in China [215], Italy [320] and Mauritius [321]. In China, triple *Wolbachia*-infected male *Ae. albopictus* were released on two small islands in Guangzhou using a combined SIT-IIT approach and the target population was suppressed with more than 99%. In Italy, the adult female populations were suppressed with 60% to 80% and this variation depended on the release density of sterile males. In Mauritius, the weekly release of sterile males suppressed the target population (in Panvati) with more than 60% for one year.

In Brazil, a new release system mounted on an unmanned aerial vehicle (UAV) or drone was tested for the aerial release of male *Ae. aegypti*. Approximately 160,000 sterile males were released over a 20 ha rural area close to Juazeiro over two weeks. A maximum sterile to wild male ratio of 0.8:1 was obtained in the release area resulting in a more than 50% of unviable eggs collected in the release area which corresponded to a *C* value of 0.26 [318].

The SIT is not a stand-alone technology, also for mosquitoes, and must be combined with other suppression tools such as source reduction (removal of larval sites) through environmental management or larvicide treatment that are particularly efficient in combination with the SIT. The use of biocides or biopesticides applied on the sterile males before release [322,323] has also been proposed to boost the impact of the method, potentially increasing the impact of SIT on mosquito populations [309,310]. This approach is currently being tested in the field.

## 7. Technology Transfer

The ultimate objective of the research and development carried out at the IPCL is to provide the basis for the development and optimization of the SIT package for selected insect pests and its transfer to operational field projects. This also includes the development of the SIT package for new species as was recently done for the Spotted Wing Drosophila *Drosophila suzukii*, the olive fruit fly *Bactrocera oleae* and the mosquitoes *Ae. aegypti and Ae. albopictus*. For other insect pests that are not being maintained at the IPCL, such as the European grapevine moth *Lobesia botrana*, the SIT package has been developed in collaboration with other research institutes.

Most of the new developments are being transferred under the umbrella of the IAEA’s Department of Technical Cooperation (IAEA TC), but this is mainly limited to developing countries. Other member states that cannot not benefit of the IAEA-TC mechanism can likewise count on IPCL support (e.g., the recent support to Lee County for the development of mosquito SIT).

The transfer of the SIT technology to the member states follows rigorously the PCA and includes technical guidance on the collection of essential baseline data, implementation of monitoring and surveillance networks, design of mass-rearing facilities, development and transfer of insect strains, support to the mass-rearing techniques, quality control, irradiation, handling and release protocols, design and support to insect emergence and release facilities, and monitoring protocols. This transfer of technology is facilitated through the availability of normative materials, such as manuals, guidelines, standard operation procedures, animated infographics, e-learning courses, leaflets, brochures, video clips, and books. All components of the SIT not discussed here have been addressed in the recently updated books on the principles and practice of the SIT [7] and the development and application of area-wide integrated pest management [324]. In addition to the above, the IPCL is hosting fellows from the different member states who receive hands-on training of all aspects of the SIT package. Those that cannot be trained at the IPCL receive training at numerous collaborating research institutes and operational programs around the word.

The importance of feedback mechanisms cannot be over emphasized. The bottlenecks and problems experienced during the implementation of such operational programmed need to be recorded and communicated to the IPCL, so that adaptive research can be carried out to solve them. In case the IPCL is not in a position to carry out this work, it will be delegated to other research institutes. This continuous feedback from operational programs is crucial and a prerequisite to continuously improve the cost-effectiveness and efficiency of the SIT.

## 8. Coordinated Research Projects

In addition to the operational or demand driven research activities carried out at the IPCL, the IPC subprogram supports research activities that are implemented through Coordinated Research Projects (CRPs). These CRPs bring together researchers from both developing and developed member states to collaborate on a research topic or problem of common interest that can contribute to the development and improvement of SIT programs. Research and technical contracts (which have a financial support) and research agreements (without any financial benefit) are awarded to institutes in member states to facilitate the agreed upon research. Each approved CRP consists of a network of 15 to 25 research institutes that operate in coordination for five years to attain the main objective of the CRP. For each contract or agreement, one institute staff member is designated as the Chief Scientific Investigator (CSI) responsible for the progress of the research work. The IAEA acts as the sponsoring and coordinating body, with an IAEA technical staff member assigned to lead each CRP as the project officer. The knowledge gained through each CRP is normally disseminated through a publication of a special issue in a peer reviewed journal. The scope of each CRP of the IPC Subprogram reflects the scientific needs that are required to solve major bottlenecks with respect to AW-IPM and the SIT application (Table 1).

## 9. Challenges and Future Prospects

### 9.1. Challenges

Despite the many advances made in research related to improve the SIT, it cannot be denied that AW-IPM programs with an SIT component remain management intensive and technically challenging [177]. To reduce the probability of failure, it is critical that programs adhere to a PCA [319,325] or the progressive control pathway (PCP) in cases where diseases are involved [326]. This ensures that the program only progresses into the next phase when all or most of the activities in the previous phase have been completed. Program implementation according to the PCA will reduce the probability of failures like the New World screwworm program in Jamaica, the tsetse project in Ethiopia or the Mediterranean fruit fly project in Egypt [177]. In view of the overall demand for reduced insecticide use and more environment-friendly insect pest control tactics, it is likely that the demand for AW-IPM approaches with an SIT component will increase in the decade to come. However, the SIT will have to compete with other genetic control tactics such as the use of transgenic strains (e.g., RIDL), gene drive techniques, Incompatible Insect Technique, just to name a few. The latter however require a regulatory framework and are in many countries still not accepted [210,211].

Increased movement of people and commodities, people and animals, combined with global warming of the climate are arguably the biggest challenges that mankind is currently facing. A direct consequence of this globalization is the increase of introductions of exotic insect species and their establishment in areas where they could not survive before. A textbook example is the establishment and spread of *Ae. albopictus* in Europe over the last three decades. The SIT, unlike insecticides and other control methods, acts in an inverse density-dependent manner, and as a result, increases its efficiency with decreasing population density [10]. Therefore, it is particularly well-suited to eliminate incipient invasive pest introductions and outbreaks when applied as part of AW-IPM approaches [324]. Examples of the use of SIT against invasive insect pests in the past are the eradication of the cactus moth *Cactoblastis cactorum* (Berg) from Mexico [327], the painted apple moth *Teia anartoides* Walker from New Zealand [328], the outbreak of the New World screwworm *Cochliomyia hominivorax* (Coquerel) from Florida, US, and the outbreak of Mediterranean fruit fly in the Dominican Republic [329] and Mexico. It will be very challenging, if not impossible, to predict where future introductions will happen, and which species will be involved. The focus could be on species that are considered highly risky and that have high potential of invasiveness. This indicates that the SIT might need to be developed for species that are considered highly invasive, e.g., the false codling moth. Another aspect is the expansion of the SIT application to include other insect pests, as has been seen recently with the development of the SIT for *D. suzukii* and the European grape vine moth *Lobesia botrana*.

### 9.2. Future Research Activities

To address some of the aforementioned challenges, and to make the SIT more competitive, the following research areas should be considered for the next decade:

- Develop SIT package for other suitable insect species
- Develop more cost-efficient mass-rearing techniques
- Establish efficient systems of colony management
- Use of endosymbionts as probiotics to improve the rearing of key insect pests and to enhance mating performance of the sterile males
- Use of semiochemicals and juvenile hormones to enhance mating performance of fruit fly species
- Fine tuning the radiation biology of different species—effects of different ionizing radiation, radiation dose rate, hypoxia, dose fractionating etc.
- Develop improved and more cost-efficient insect release systems using aircraft, gyrocopters or drones
- Unravel the relationships between different endosymbionts and pathogens to enhance male performance, or develop sterile males (in the case of tsetse flies) that are refractory to transmitting pathogens
- Develop more efficient genetic sexing systems, especially for human disease vectors

## Figures and Tables

**Table 1:** Coordinated research projects that have been implemented in the last 10 years, and special issues that were published to disseminate the results.

Project Number	Coordination Research Projects	Special Issue (Reference)
G3.40.01	Development of Standardized Mass Rearing Systems for Male Mosquitoes (2005–2011)	International Journal of Tropical Insect Science|Volume 34, supplement issue 1 (springer.com)
D4.20.12	Improving SIT for Tsetse Flies through Research on their Symbionts and Pathogens (2007–2012)	Journal of Invertebrate Pathology|Tse Tse Fly Symposium|ScienceDirect.com by Elsevier
G3.40.02	Biology of Male Mosquitoes in Relation to Genetic Control Programs (2008–2013)	Acta Tropica|Biology and behaviour of male mosquitoes in relation to new approaches to control disease transmitting mosquitoes|ScienceDirect.com by Elsevier
D4.20.13	Applying Population Genetics and GIS for Managing Livestock Insect Pests (2008–2013)	Acta Tropica|Applying GIS and population genetics for managing livestock insect pests: Case studies of tsetse and screwworm flies|ScienceDirect.com by Elsevier
D4.10.22	Increasing the Efficiency of Lepidoptera SIT Through Enhanced Quality Control (2009–2014)	Volume 99 Special Issue 1|Florida Entomologist (bioone.org)
D6.20.08	Development of Generic Irradiation Doses for Quarantine Treatments (2009–2014, managed by Food and Environmental Protection Subprogram)	Vol. 99, Special Issue 2 (October 2016)|Florida Entomologist (flvc.org)
D4.20.14	Development and Evaluation of Improved Strains of Insect Pests for SIT (2009–2014)	BMC Genomic Data|Volume 15, supplement issue 2 (springer.com)
D4.20.23	Resolution of Cryptic Species Complexes of Tephritid Pests to Overcome Constraints to SIT Application and International Trade (2010–2015)	Resolution of Cryptic Species Complexes of Tephritid Pests to Enhance SIT Application and Facilitate International Trade (pensoft.net)
D4.10.24	Use of Symbiotic Bacteria to Reduce Mass-Rearing Costs and Increase Mating Success in Selected Fruit Pests in Support of SIT Application (2012–2017)	Proceedings of an FAO/IAEA coordinated research project on use of symbiotic bacteria to reduce mass-rearing costs and increase mating success in selected fruit pests in support of SIT application (biomedcentral.com)
D4.20.15	Enhancing Vector Refractoriness to Trypanosome Infection (2013–2018)	BMC Microbiology|Enhancing vector refractoriness to trypanosome infection (biomedcentral.com)
D4.40.01	Exploring Genetic, Molecular, Mechanical and Behavioral Methods of Sex Separation in Mosquitoes (2013–2018)	Parasites & Vectors|Exploring genetic, molecular, mechanical and behavioural methods of sex separation in mosquitoes (biomedcentral.com)
D4.20.16	Comparing Rearing Efficiency and Competitiveness of Sterile Male Strains Produced by Genetic, Transgenic or Symbiont-based Technologies (2015–2020)	BMC Genomic Data|Comparing rearing efficiency and competitiveness of sterile male strains produced by genetic, transgenic or symbiont-based technologies (biomedcentral.com)
D4.10.25	Dormancy Management to Enable Mass-rearing and Increase Efficacy of Sterile Insects and Natural Enemies (2014–2019)	In preparation
D4.40.02	Mosquito Handling, Transport, Release and Male Trapping Methods (2015–2020)	In preparation
D4.10.26	Improved Field Performance of Sterile Male Lepidoptera to Ensure Success in SIT Programmes (2016–2021)	-
D4.30.03	Integration of the SIT with Biocontrol for Greenhouse Insect Pest Management (2017–2022)	-
D4.20.17	Improvement of Colony Management in Insect Mass-rearing for SIT Applications (2018–2023)	-
D4.10.27	Assessment of Simultaneous Application of SIT and MAT to Enhance *Bactrocera* Fruit Fly Management (2019–2024)	-
D4.40.03	Generic Approach for the Development of Genetic Sexing Strains for SIT Applications (2019–2024)	-
D4.40.04	Mosquito Radiation, Sterilization and Quality Control (2020–2025)	-

## Data Availability

This is a review paper and all original data can be found in the cited publications.
